# Universal Mindfulness Training in Schools for Adolescents: a Scoping Review and Conceptual Model of Moderators, Mediators, and Implementation Factors

**DOI:** 10.1007/s11121-022-01361-9

**Published:** 2022-03-10

**Authors:** Kate Tudor, Shannon Maloney, Anam Raja, Ruth Baer, Sarah-Jayne Blakemore, Sarah Byford, Catherine Crane, Tim Dalgleish, Katherine De Wilde, Tamsin Ford, Mark Greenberg, Verena Hinze, Liz Lord, Lucy Radley, Emerita Satiro Opaleye, Laura Taylor, Obioha C. Ukoumunne, Russell Viner, MYRIAD Team, Willem Kuyken, Jesus Montero-Marin

**Affiliations:** 1 Department of Psychiatry, University of Oxford, Oxford, UK; 2 Faculty of Public Health and Policy, London School of Hygiene and Tropical Medicine, London, UK; 3 Department of Psychology, University of Cambridge, Cambridge, UK; 4 Health Service and Population Research, King’s College London, London, UK; 5 MRC Cognition and Brain Sciences Unit, University of Cambridge, Cambridge, UK; 6 Department of Psychiatry, University of Cambridge, Cambridge, UK; 7 Human Development and Family Studies, Penn State University, State College, USA; 8 Departamento de Psicobiologia, Universidade Federal de São Paulo, São Paulo, Brazil; 9 NIHR ARC Southwest Peninsula, Exeter Medical School, Exeter, UK; 10 Institute of Child Health, University College London, London, UK; 11 Teaching, Reseach & Innovation Unit, Parc Sanitari Sant Joan de Déu, Sant Boi de Llobregat, Spain

**Keywords:** Mindfulness, School-based programmes, Adolescence, Universal interventions, Prevention programmes, Mediators, Moderators, Implementation

## Abstract

There is evidence that universal school-based mindfulness training (SBMT) can have positive effects for young people. However, it is unknown who benefits most from such training, how training exerts effects, and how implementation impacts effects. This study aimed to provide an overview of the evidence on the mediators, moderators, and implementation factors of SBMT, and propose a conceptual model that can be used both to summarize the evidence and provide a framework for future research. A scoping review was performed, and six databases and grey literature were searched. Inclusion and exclusion criteria were applied to select relevant material. Quantitative and qualitative information was extracted from eligible articles and reported in accordance with PRISMA-ScR guidelines. The search produced 5479 articles, of which 31 were eligible and included in the review. Eleven studies assessed moderators of SBMT on pupil outcomes, with mixed findings for all variables tested. Five studies examined the mediating effect of specific variables on pupil outcomes, with evidence that increases in mindfulness skills and decreases in cognitive reactivity and self-criticism post-intervention are related to better pupil outcomes at follow-up. Twenty-five studies assessed implementation factors. We discuss key methodological shortcomings of included studies and integrate our findings with existing implementation frameworks to propose a conceptual model. Widespread interest in universal SBMT has led to increased research over recent years, exploring who SBMT works for and how it might work, but the current evidence is limited. We make recommendations for future research and provide a conceptual model to guide theory-led developments.

**Supplementary Information:**

The online version contains supplementary material available at 10.1007/s11121-022-01361-9.

## Introduction

Mental health problems commonly have their first onset in adolescence, which is a period of increased vulnerability associated with reduced attentional, behavioural, and emotional regulation (Blakemore, 2008; Paus et al., 2008). Around 50% of all mental health problems appear before the age of 14 years (Kessler et al., 2005). Not only is this associated with persistent social, emotional, and behavioural problems in adolescence, it also predicts mental health difficulties in adulthood (Greenberg & Abenavoli, 2017; Solmi et al., 2021). Hence, the development of programmes for adolescents to reduce risk of mental ill health and promote well-being is crucial.

Universal preventive approaches have gained traction for improving mental health in young people (Fusar-Poli et al., 2021). By aiming to reduce risk factors that are shared amongst multiple mental health conditions, whilst promoting protective factors that can facilitate coping across settings and experiences, universal mental health promotion holds great potential for reducing risk at the population scale (Greenberg & Abenavoli, 2017). Schools play a central role in the lives of young people and families. They provide an opportune setting for promoting mental health since kids spend so much time there and programmes can be implemented as part of a preventive approach at relatively low cost per person, compared to more intensive and targeted interventions (Greenberg, 2010; Weare & Nind, 2011). By teaching foundational skills, such as attention and engagement in learning, school-based universal approaches may support a broad range of outcomes, including academic performance and well-being (Vostanis et al., 2013). Examples of school-based universal mental health promotion include social and emotional learning (SEL) and school-based mindfulness training (SBMT).

SEL programmes are focussed on helping individuals manage their emotional states, reach goals with empathy for others, maintain positive relationships, and make responsible decisions. These programmes have demonstrated improvement in students’ skills, attitudes, and social behaviours (Durlak et al., 2011). Conversely, SMBT is mainly focussed on training awareness and cognitive processes that are central to mental health and academic performance, as well as values, caring for others, and personal growth, and has only recently been implemented in school contexts. Both SEL and SBMT try to develop positive facets of the self, including moral, social, and emotional aspects (Lawlor, 2016). However, arguably one key distinction between SEL and SBMT programmes is that SBMT involves the practice of mindfulness, which entails cultivating present-moment awareness of one’s experience (Bishop et al., 2004).

Mindfulness-based programmes were introduced in mainstream settings when mindfulness-based stress reduction (MBSR) and mindfulness-based cognitive therapy (MBCT) were developed to treat chronic pain and recurrent depression respectively. Both MBSR and MBCT are informed by science, education, and contemplative practices (Crane et al., 2017); teach foundational skills of attention and self-regulation; and, if taught well, are non-stigmatizing (Crane et al., 2017). Evidence indicates that these programmes are effective in improving a number of outcomes in adults, including depression (Goldberg et al., 2018; Khoury et al., 2013), risk of relapse for depression (Kuyken et al., 2016), stress (Khoury et al., 2013), anxiety (Khoury et al., 2013), and sleep quality (Rusch et al., 2019). Given the benefits of training for adults, universal adaptations of mindfulness programmes have been developed for school settings. Interest in universal SBMT has increased over the past decade and there is promising evidence from randomised controlled trials (RCTs) that they reduce symptoms of depression, anxiety, and stress in adolescents (Baelen et al., 2019; Dunning et al., 2019; Roeser et al., 2020). However, there is little evidence that SBMT reduces negative behaviours (e.g., aggression) when compared with active controls, nor that it works through its hypothesized mechanisms (Dunning et al., 2019; Roeser et al., 2020).

Trials of universal SBMT may not capture the full range of effects because benchmarks of effectiveness prioritise individual-level rather than population-level impacts, and mean impact across the trial rather than subgroup effects (Greenberg & Abenavoli, 2017). Thus, more effort is needed to consolidate the evidence on why universal SBMT works (i.e. through which mechanisms), what proximal and distal outcomes SBMT influences, and for whom SBMT may work best (i.e. which adolescents receive greatest or least benefit).

### Process Evaluations in SBMT

Guidelines recommend that evaluations of complex interventions such as SBMT should include modelling of outcomes and moderators that may explain discrepancies between expected and observed results (Craig et al., 2019). An example in school-based programmes might be characteristics of students or the school that may differentially impact outcomes. Another consideration is to understand how SBMT works. We need to disentangle the processes through which the programme produces its effects (i.e. mechanisms of change). To understand how SBMT exerts effects, mediation analyses are required. Ultimately, an understanding of both moderators and mediators is important if we have the goal of improving SBMT. Examining moderators allows us to know for whom this kind of training is adequate, and identifying mediators illuminates the interim processes between the SBMT and the outcome, facilitating the reinforcement of those aspects of the programme that are functioning as pathways of change. In addition to moderation and mediation, quality of implementation is also an important consideration when studying universal prevention programmes. It has been observed that implementation factors impact the efficacy of school-based SEL programmes. A review of 213 universal SEL programmes demonstrated that the presence of implementation problems substantially reduced effect sizes (Durlak et al., 2011). In the case of SBMT, it has been suggested that differences across studies in terms of the implementation might account for mixed findings regarding efficacy (Emerson et al., 2020). Implementation refers to the execution of an evidence-based programme in practice. Five main aspects of implementation include fidelity (the extent to which the delivered programme corresponds to the original programme); dosage (how much of the original programme has been received); quality (how well different programme components are delivered); participant responsiveness (the degree to which the programme engages and stimulates the interest of participants); and programme differentiation (the extent to which programme theory and practices can be distinguished from other programmes) (Dane & Schneider, 1998; Durlak & DuPre, 2008). Additionally, Durlak and DuPre (2008) have proposed three more aspects: monitoring of the control group (e.g. treatment contamination), programme reach (e.g. participation rates with respect to the study population), and adaptation of the programme.

There are previous systematic and narrative reviews that have been published in the last 10 years on SBMT programmes, and some of them have touched on mediators and issues of implementation (Emerson et al., 2020; Felver et al., 2016; Greenberg & Harris, 2012, McKeering & Hwang, 2019; Meiklejohn et al., 2012; Zenner et al., 2014). However, none of them has developed a general explanatory conceptual model, integrating potential moderators, mediators, and implementation factors that could serve as a roadmap for future research and developments in the SBMT field.

### Study Aims

This paper presents a comprehensive scoping review. We summarise the literature on frequency and impact of moderators, mediators, and implementation factors on SBMT outcomes for adolescents, and we enumerate the outcomes these studies considered. Based on theory, extant research, and the scoping review, we then develop an explanatory conceptual model to serve as a guide for future research.

## Methods

### Scoping Review Methodology

This scoping review was conducted in accordance with the methodology of the Joanna Briggs Institute’s Manual (Peters et al., 2020), and is reported following the Preferred Reporting Items for Systematic reviews and Meta-Analyses extension for scoping reviews ‒ the PRISMA-ScR Statement (Tricco et al., 2018). The protocol was registered on Open Science Framework on 8 October 2020 (https://osf.io/wahet). We considered an intervention SBMT if the core of the programme was focussed on developing mindfulness skills. Since publishing the protocol, we made two changes: the decision to include Spanish references (due to language skills of the research team), and to synthesise data relating to pupil outcomes only (rather than pupil and teacher outcomes) to help narrow the focus. Scoping reviews are similar to systematic reviews in that they follow a structured process, but they are performed for different objectives and have methodological differences. A scoping review design was chosen here as the aim was to map out the available evidence, to examine how key concepts and definitions within the literature are used, and to identify knowledge gaps and future avenues.

### Search Strategy

The research team collated a broad list of terms pertinent to school-based mindfulness research. Terms relating to participants (e.g. adolescents), context (e.g. school), and concepts (e.g. mediators, moderators, and implementation factors) were generated. The complete search strategy is presented in Online Resource 1. The following six databases were searched: PubMed, PsycINFO, Embase, Scopus, Cochrane Central, and ERIC. Databases were searched from inception up until 12 October 2020 and the search was restricted to those in English and Spanish languages due to the first languages of the research team. The search was re-run on 10 April 2021. Forward citation tracking and reference checking from included studies were performed. Finally, reference lists of relevant literature (e.g. book chapters, systematic reviews, meta-analyses, and conference proceedings) and Google Scholar were also hand searched.

### Inclusion and Exclusion Criteria

Articles had to meet the following criteria: (1) evaluated universal SBMT aimed at school-attending adolescents; (2) evaluated mindfulness training that was implemented as part of the school curriculum; (3) evaluated mindfulness training as a core component of the programme; (4) evaluated moderators, mediators, or implementation factors, quantitatively. It was not required for authors to explicitly state what they were exploring. If reviewers of the research team inferred that findings discussed moderators, mediators, or implementation factors, then the study was considered for inclusion.

Articles were excluded for the following reasons: (1) evaluated mindfulness training exclusively targeted towards at-risk populations; (2) evaluated mindfulness training delivered outside the school curriculum; (3) evaluated training where mindfulness practice was not a core component; (4) students under the age of 11 years were excluded due to developmental differences confounding the effect of SBMT on outcomes. If studies included students from both primary (elementary) and secondary (high) school, and analyses did not split by age group, then they were excluded.

### Selection, Data Charting, and Synthesis of Results

All titles and abstracts were screened by two reviewers independently (RB, SM, JMM, LR, AR, KT), with discrepancies resolved by a third reviewer or group discussion. Only clearly irrelevant references were excluded at this stage. Full texts were obtained for all potentially relevant references. Where references were not available, the corresponding author was contacted. Full texts were reviewed for eligibility by two reviewers, with uncertainties resolved by a third party.

For included references, four reviewers (SM, JMM, LR, KT) extracted the following characteristics: study sample, school characteristics, recruitment methods, programme characteristics (e.g. setting, duration, frequency of sessions, components), programme quality (e.g. training of teachers), outcome measures, analysis of moderators, analysis of mediators, measure of implementation, and general findings. We did not limit outcome measures and any outcome measures reported following SBMT were extracted. The data extraction form was first piloted with a subset of references by two reviewers (AR, KT) prior to full extraction.

The documents were categorised according to their evaluation of moderators, mediators, or implementation. Outcomes were classified by five categories: (1) mindfulness and self-regulation skills; (2) mental health; (3) physical health; (4) healthy relationships with others and the physical world; and (5) school behaviour and academic performance to mirror Roeser et al. (2020). A narrative synthesis was conducted.

### Conceptual Model Development and Methodology

We used extant theory and research describing how SBMT factors, across the pupil, teacher, and wider context levels, interact to promote change (Bergström et al., 2020; Durlak & DuPre, 2008; Jennings & Greenberg, 2009; Meyers et al., 2012; Munn et al., 2018; Roeser et al., 2013), together with the findings of the scoping review, to develop a conceptual model that can be used to summarize how potential moderators, mediators, and implementation factors of SBMT fit together as a theory of change. Additionally, we used existing implementation frameworks–e.g. the Durlak Framework, Quality Implementation Framework, the PARIHS framework, and the Medical Research Council guidance for developing and evaluating complex interventions (Bergström et al., 2020; Meyers et al., 2012; Moore et al., 2015) –to inform implementation factors in the model. The process of distilling the model was based on content analysis of the categories that were included in the extant research and implementation frameworks. Following the constant comparative method (Glaser & Strauss, 1967), two researchers made their classification of themes until a common conceptual denomination was used for all the frameworks. A third researcher was in charge of resolving possible disagreements. The possible relations between the themes were clearly expressed to represent a highly parsimonious solution, which enabled the emerging conceptual structure to gain density.

## Results

**Fig. 1:**
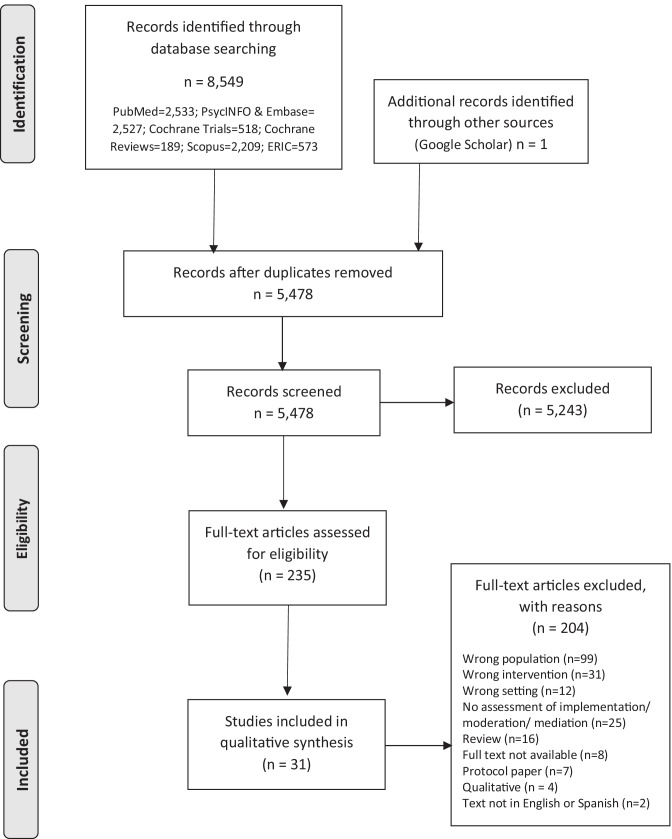
Flow diagram of scoping review process

The search of electronic databases and hand searches of grey literature generated a list of 8549 titles and abstracts. Of these, 5478 titles and abstracts were eligible for screening after removing duplicates. Of these, 235 were considered for full text review (see Fig. 1). Thirty-one articles (all of them published in English) were included in the narrative synthesis. The characteristics of all included articles are shown in Table 1 and Online Resource 2.

**Table 1:** General overview of included studies

Author (year)	Age (years)	Intervention	Design	Time-points	Setting	*n*	Mod	Med	Imp	Outcomes
Anand and Sharma (2011)	14.23 (NR)	MBSR modified	Single arm	2 (pre, 3 months post)	Class	35	No	No	Yes	Stress, well-being
Atkinson and Wade (2015)	15.7 (0.77)	Mindfulness	RCT	3 (pre, 1 and 6 months post)	Class	347	Yes	No	Yes	Weight-shape concern, negative affect, dietary restraint, thin-ideal internalization, and socio-cultural pressures, eating disorder symptoms, psychosocial impairment
Bauer et al. (2020)	11.7 (0.40)	Mindfulness	RCT	2 (pre and post)	Class	40	No	No	Yes	Sustained attention, functional brain connectivity
Bergen-Cico et al. (2015)	11.4 (0.03)	Mindful yoga		3 (pre, mid, post)	Class	144	No	No	Yes	Self-regulation
Britton et al. (2014)	11.8 (0.41)	Mindfulness	Mixed method (RCT and qualitative)	3 (baseline, pre, post)	Class	101	No	Yes	Yes	Behavioural and emotional problems, anxiety, mindfulness
Broderick and Frank (2014)	17.43 (0.53)	Mindfulness	Quasi-experimental	2 (pre, post)	Class		No	No	Yes	(No outcomes are included)
Butzer et al. (2017)	12.64 (0.33)	Mindfulness and yoga	RCT	4 (pre, 1 week, 6 months and 1 year post)	PhE	211	Yes	No	Yes	Emotion regulation, stress, mood impairment, impulsivity, substance use
Campbell (2015)	15.96 (1.17)	Mindfulness	Quasi-experimental	2 (pre, post)	Class	1007	Yes	No	No	Stress, experiences in close relationships, emotion regulation, positive and negative affect
Chancey (2018)	14.93 (0.66)	Mindfulness and yoga	RCT	3 (baseline, 1 week, 14 weeks post)	PhE	80	No	No	Yes	Stress, emotion regulation, mindfulness, anxiety, depression, academic efficacy, disruptive behaviour, school attendance, disciplinary infractions
Clarke et al. (2021)	15.2 (1.3)	Mindfulness and yoga	RCT	2 (pre, 1 month post)	PhE	187	No	No	Yes	Stress, self-regulation, mindfulness, anxiety, depression
Daly et al. (2015)	16 (NR)	Mindfulness and yoga	RCT	2 (pre, post (> 2 weeks))	PhE	38	No	Yes	No	Emotion regulation, mindfulness, self-compassion, interoceptive awareness
Frank et al. (2021)	16 (NR)	MBSR modified	RCT	2 (pre, post)	Class	255	No	No	Yes	Depression, anxiety, rumination, stress, somatization, sleep, emotion regulation, mindfulness, self-compassion, social connectedness, mind-wandering, mindset, substance use
Huppert and Johnson (2010)	Range 14–15	MBSR modified	RCT	2 (pre, post)	Class	134	Yes	No	Yes	Mindfulness, resilience, well-being, personality
Johnson et al. (2016)	13.63 (0.43)	MBSR modified	Mixed method (RCT and qualitative)	3 (1 week pre- and post, 11 weeks post)	Class	308	Yes	No	Yes	Anxiety, depression, weight/shape concerns, well-being, emotional dysregulation, self-compassion, mindfulness
Johnson et al. (2017)	13.44 (0.33)	MBSR modified	RCT	4 (3–4 weeks pre, post and 6- and 12-months post)	Class	555	Yes	No	Yes	Anxiety, depression, weight/shape concerns, well-being, mindfulness
Johnson and Wade (2019)	14.28 (1.08)	MBSR modified	Quasi-experimental	3 (pre, post, 4 months post)	Class	90	No	No	Yes	Depression, anxiety, well-being, weight/shape concerns
Kang et al. (2018)	11.8 (0.41)	Mindfulness	RCT	2 (pre and post)	Class	100	Yes	No	No	Anxiety, mindfulness, self-compassion
Khalsa et al. (2012)	16.80 (0.60)	Mindfulness and yoga	RCT	2 (pre and post)	PhE	121	No	No	Yes	Personality, mood disturbance, anxiety, stress, resilience, positive attitudes
Kuyken et al. (2013)	14.9 (1.5) (int only)	Mindfulness	Quasi-experimental	3 (baseline, post, and 3 months after baseline)	Class	522	No	No	Yes	Well-being, stress, depression
Lam and Seiden (2020)	12.4 (NR)	Mindfulness (brief)	Quasi-experimental	2 (pre and post)	Class	115	No	No	Yes	Stress, emotion regulation, rumination, internalizing and attention problems, executive function
Lawson (2019)	NR Grade 9 (USA)	Mindfulness	Mixed method (single arm and qualitative)	2 (pre and post)	Class	50	No	No	Yes	Mindfulness, student engagement, school climate (teacher rated)
Lombas et al. (2019)	13.6 (1.5)	Mindfulness	Quasi-experimental	2 (pre, 6 months post)	Class	524	Yes	Yes	Yes	Mindfulness, well-being, depression, stress, psychological needs, emotional intelligence, aggression, classroom climate, academic motivation, empathy
López-González et al. (2018)	14.29 (1.52)	Mindfulness	Single arm trial	2 (pre and post)	Class	420	No	Yes	No	Mindfulness, relaxation, classroom climate, academic performance
Metz et al. (2013)	16.5 (0.9)	MBSR modified	RCT	2 (pre and post)	Class	244	No	No	Yes	Emotion regulation, psychosomatic complains, stress, self-efficacy in emotion regulation
Mrazek et al. (2019)	NR Grade 10–12	Mindfulness	Mixed method (single arm and Qualitative)	2 (pre and post)	Class (online)	346	No	No	Yes	Emotion regulation, life satisfaction, mind-wandering, mindset about focus, life demands, stress, stress management, classroom distraction
Rice et al. (2015)	Range: 13–14	MBCT modified	Quasi-experimental	2 (pre and 9 weeks post)	Class	256	Yes	No	Yes	Depression, reward-processing, negative self-beliefs, autobiographical memory
Salmoirago-Blotcher et al. (2018)	14.6 (0.3)	MBSR modified	RCT	2 (pre and 6 months post)	Class	53	Yes	No	Yes	Physical activity, dietary habits
Sibinga et al. (2016)	12 (NR)	MBSR modified	RCT	2 (pre and post)	Class	300	No	No	Yes	Mindfulness, depression, paranoid ideation, hostility, somatization, anxiety, positive and negative affect, emotion regulation, aggression, anger, coping, posttraumatic symptoms
Van der Gucht et al. (2017)	15.4 (1.1)	MBSR modified	RCT	3 (pre, post, follow-up)	Class	553	Yes	No	No	Depression
Van der Gucht et al. (2018)	15.4 (1.2)	MBSR modified	RCT	3 (pre, post, follow-up)	Class	408	No	Yes	No	Depression, anxiety, stress, cognitive reactivity, self-compassion
Worthen and Luiselli (2019)	NR	Mindfulness	Quasi-experimental	2 (pre and post)	School	86	No	No	Yes	(Only mindfulness practice measures are included)

Age-related figures are means (standard deviations). Additional details of included studies can be found in Online Resource 2

*Mod* moderation analysis, *Med* mediation analysis, *Imp* implementation analysis, *PhE* physical education, *NR* not reported, *n* sample size, *MBSR* Mindfulness-Based Stress Reduction, *MBCT* Mindfulness-Based Cognitive Therapy, *RCT* randomised controlled trial

We did not limit our search for specific outcome variables and thus report studies that include an examination of mediators, moderators, or implementation factors in relation to any outcome. Outcomes pertained to mindfulness (13 studies), stress (13 studies), depression (12 studies), anxiety (11 studies), self-regulation (12 studies), well-being (7 studies), self-compassion (5 studies), weight/shape concerns (4 studies), affect (3 studies), school/classroom climate (3 studies), substance use (2 studies), and resilience (2 studies). Other less frequent outcomes can be seen in Table 1.

### What Moderates the Effect of SBMT on Outcomes?

Eleven studies assessed potential moderators of SBMT (Online Resource 3) (Atkinson & Wade, 2015; Butzer et al., 2017; Campbell, 2015; Huppert & Johnson, 2010; Johnson et al., 2016, 2017; Kang et al., 2018; Lombas et al., 2019; Rice et al., 2015; Salmoirago-Blotcher et al., 2018; Van der Gucht et al., 2017). Moderation analysis methods varied and included linear mixed-effects regression, multiple linear regression, and split-plot ANOVAs. One study found efficacy on depression, stress, competence, emotional intelligence, and academic motivation only when pre-treatment mindfulness was high (Lombas et al., 2019). Six studies tested gender as a moderator; three indicated that girls showed greater benefit from SBMT in improving emotional regulation (Butzer et al., 2017), anxiety (Johnson et al., 2016), and positive affect (Kang et al., 2018); one indicated that boys showed greater benefit in adhering to physical activity (Salmoirago-Blotcher et al., 2018); two reported no significant gender effects on depression (Johnson et al., 2017; Van der Gucht et al., 2017); and one obtained no significant gender effects on anxiety, weight/shape concerns, well-being, and mindfulness (Johnson et al., 2017). Two studies tested age as a moderator; one indicated older adolescents showed greater benefit from SBMT compared to younger adolescents on depression (Van der Gucht et al., 2017); and one showed no significant effects of age on depression, anxiety, weight/shape concerns, well-being, and mindfulness (Johnson et al., 2017). Four studies assessed baseline mental health status as a potential moderator; one showed that adolescents with poorer mental health showed greater benefits on depression (Van der Gucht et al., 2017), while two showed no significant mental health effects on weight/shape concerns, negative affect, dietary restraint, thin-ideal internalization, socio-cultural pressures, eating disorder symptoms, and psychosocial impairment (Atkinson & Wade, 2015), as well as depression, anxiety, weight/shape concerns, well-being, and mindfulness (Johnson et al., 2017); and one demonstrated that anxiety got worse for those with lower baseline levels of weight/shape concerns or depression (Johnson et al., 2016). One study found that students with high attachment anxiety experienced greater declines in negative affect than those with low attachment anxiety (Campbell, 2015); and one study observed that higher agreeableness and lower emotional stability were associated with greater improvements in well-being (Huppert & Johnson, 2010). One study found no significant moderating effects of cognitive variables such as reward-seeking, negative self-beliefs, or autobiographical memory on depression (Rice et al., 2015). Finally, one study found no significant effect of school-type (vocational, technical, or general education) on the effects of a SBMT on depression in the context of the Belgian school system (Van der Gucht et al., 2017).

### What Mediates the Effect of SBMT on Outcomes?

Five studies examined mediators of SBMT on outcomes (Online Resource 3) (Britton et al., 2014; Daly et al., 2015; Lombas et al., 2019; López-González et al., 2018; Van der Gucht et al., 2018). Methods of mediation analyses included correlation analysis, Sobel’s mediation test, Hayes’ (2013) mediation test, and lower level time-lagged mediation modelling. Lombas et al. (2019) tested whether improvements in mindfulness skills mediated the effect of SBMT on multiple mental health and behavioual outcomes. Improvements on well-being, emotional disturbance, competence, relationships, emotional attention, aggression, teacher support, motivation, and empathy at follow-up were mediated by increases in mindfulness skills (Lombas et al., 2019). In this same line, one study observed that improvements in mindfulnes skills were significantly correlated with reductions in affect disturbance, and with increases in positive affect (Britton et al., 2014). In contrast, another study aimed to test the potential mediating role of changes in mindful awareness and self-compassion between pre-post SBMT on emotion regulation at post-test. However, neither variable was correlated with emotion regulation and therefore mediation analyses were not conducted (Daly et al., 2015).

One study tested whether changes in cognitive reactivity mediated the effect of SBMT on depression, anxiety, and stress (Van der Gucht et al., 2018). Mediation analyses indicated that decreases in cognitive reactivity mediated the reduction in symptoms of depression, anxiety, and stress. López-González et al. (2018) examined the mediating role of change in classroom climate on the relationship between SBMT and improved academic performance, with no signifficant mediation reported. It is important to note that there are limitations of these studies reporting mediation. To adequately assess mediation, three time points are required as mediators must temporally precede outcomes rather than measured at the same time (Kazdin, 2007). Of the four studies exploring mediators, three measured mediators at the same time as outcomes. Therefore, findings can only be treated as exploratory.

### How Does Implementation Influence the Effect of SBMT on Outcomes?

Of the 31 included studies, 25 reported data relating to implementation (Online Resource 4) (Anand & Sharma, 2011; Atkinson & Wade, 2015; Bauer et al., 2020; Bergen-Cico et al., 2015; Britton et al., 2014; Broderick & Frank, 2014; Butzer et al., 2017; Chancey, 2018; Clarke et al., 2021; Frank et al., 2021; Huppert & Johnson, 2010; Johnson & Wade, 2019; Johnson et al., 2016, 2017; Khalsa et al., 2012; Kuyken et al., 2013; Lam & Seiden, 2020; Lawson, 2019; Lombas et al., 2019; Metz et al., 2013; Mrazek et al., 2019; Rice et al., 2015; Salmoirago-Blotcher et al., 2018; Sibinga et al., 2016; Worthen & Luiselli, 2019). Here, we frame descriptive information regarding implementation using the Durlak and DuPre (2008) framework, described above.

#### Dosage

Ten studies reported programme dosage, which was operationalised as the following: participant attendance to SBMT (Anand & Sharma, 2011; Butzer et al., 2017; Chancey, 2018; Khalsa et al., 2012; Lawson, 2019; Metz et al., 2013; Mrazek et al., 2019; Salmoirago-Blotcher et al., 2018; Sibinga et al., 2016), the number of days the programme was delivered (Bergen-Cico et al., 2015), and total minutes the programme was implemented for (Butzer et al., 2017). Dosage was high for studies that reported on this measure, as might be expected given levels of school attendance. Only one study – using secondary analyses– commented on the impact of dosage on outcomes, showing that a higher dose was related to increases in positive attitudes towards school and decreases in mood disturbance post-intervention (Khalsa et al., 2012).

#### Participant Responsiveness

Twenty-two of the included 31 studies reported an indicator of participant responsiveness (Anand & Sharma, 2011; Atkinson & Wade, 2015; Bauer et al., 2020; Bergen-Cico et al., 2015; Britton et al., 2014; Broderick & Frank, 2014; Butzer et al., 2017; Chancey, 2018; Clarke et al., 2021; Frank et al., 2021; Huppert & Johnson, 2010; Johnson & Wade, 2019; Johnson et al., 2016, 2017; Khalsa et al., 2012; Kuyken et al., 2013; Lam & Seiden, 2020; Lawson, 2019; Lombas et al., 2019; Metz et al., 2013; Salmoirago-Blotcher et al., 2018; Worthen & Luiselli, 2019). Authors conceptualised responsiveness differently (e.g. responsiveness, receptiveness, feedback, acceptability, satisfaction, enjoyment) and concepts were operationalised in a variety of ways (e.g. ratings of usefulness, perceived benefits, engagement, enjoyment, helpfulness, and intentions to apply to daily life). Where students were asked to rate their responsiveness on a Likert-type scale, the reported means generally fell in the middle of the scale. For most studies reporting participant responsiveness, standard deviations and ranges of scale scores indicated that pupil responses were variable. One study presented that 17% of students randomised to the programme indicate extremely negative responses to SBMT (Butzer et al., 2017). For studies that measured responses to the programme as well as intentions to use aspects of the programme in the future, mean scores were lower for future intentions of use, indicating that, while students may enjoy a training programme, this may not translate to future behaviour (Atkinson & Wade, 2015; Kuyken et al., 2013). One study explored the association between responsiveness and outcomes, finding that higher satisfaction with the prevention programme was associated with pre-post improvements in affective self-regulatory efficacy and emotional awareness (Metz et al., 2013).

The extent of pupils’ self-reported practice outside of the prescribed intervention (i.e. home practice) was measured in 12 studies (Anand & Sharma, 2011; Broderick & Frank, 2014; Butzer et al., 2017; Chancey, 2018; Frank et al., 2021; Huppert & Johnson, 2010; Johnson et al., 2016, 2017; Johnson & Wade, 2019; Lawson, 2019; Salmoirago-Blotcher et al., 2018; Worthen & Luiselli, 2019). Most studies reported low levels of home practice. Seven studies examined the influence of practice on outcomes. Four of them found a significant positive association, while three studies found no significant associations. Broderick and Frank (2014) tested whether mean gain scores for all outcome measures were associated with student self-reported home practice. Only somatic complaints were reduced for those practicing mindfulness for four or more days a week in comparison to those practicing less. Huppert and Johnson (2010) reported that students’ self-reported mindfulness practice (low [once a week] vs medium [less than 3 × a week] and high [at least three times a week]) significantly predicted changes in mindfulness and well-being, but not changes in resilience. Similarly, Kuyken et al. (2013) ran random effects linear regression models and found that students reporting more frequent use of mindfulness practices had better outcomes for well-being, depression, and stress at follow-up. Finally, Frank et al. (2021) conducted exploratory analyses of the moderating effect of home practice on outcomes, finding the programme resulted in more beneficial effects on outcomes (emotion regulation, emotional awareness and clarity, impulse control, mind wandering, social connectedness, and substance use) for students who reported practicing more than once a month compared to those practicing less than once a month. In contrast, Lam and Seiden (2020) reported that self-reported home practice was not significantly correlated with perceived stress, emotion regulation, rumination, or attention at follow-up. Similarly, two studies used linear mixed effects models to test home practice as a moderator of programme effect, finding no significant effect on mental health outcomes (Johnson et al., 2016, 2017).

#### Fidelity

Ten studies measured fidelity using either self-reported adherence checklists, self-evaluation (Chancey, 2018; Lawson, 2019; Lombas et al., 2019; Metz et al., 2013; Rice et al., 2015), or independent ratings of a proportion of programme sessions covered (Johnson & Wade, 2019; Johnson et al., 2017; Metz et al., 2013; Rice et al., 2015; Salmoirago-Blotcher et al., 2018). Two of these studies reported measuring fidelity but did not report findings (Chancey, 2018; Lawson, 2019). Most studies reported that the programme was delivered with high fidelity. However, no studies examined the influence of fidelity on outcomes.

#### Quality

Eight studies reported an indicator of programme quality (Anand & Sharma, 2011; Atkinson & Wade, 2015; Johnson & Wade, 2019; Johnson et al., 2016, 2017; Kuyken et al., 2013; Metz et al., 2013; Rice et al., 2015). Four studies used the same standardised measure (Mindfulness Based Interventions – Teacher Assessment Criteria, MBI-TAC) (Crane et al., 2013). Of these, three were studies where SBMT was delivered by an experienced external practitioner and quality ratings were completed by an independent observer (Johnson & Wade, 2019; Johnson et al., 2016, 2017). Quality ratings on the MBI-TAC for external mindfulness instructors were high. One study tested the effect of teacher status (optimally trained facilitator vs non-expert facilitator) by conducting post hoc sub-group analyses (Atkinson & Wade, 2015). Results indicated that students showed greater benefits from SBMT when taught by the optimally trained facilitator compared to the non-expert on weight and shape concerns, dietary restraint, thin-ideal internalizations, eating disorder symptoms, and psychosocial impairment.

#### Differentiation, Adaptation, Contamination of Control Group, and Pupil Reach

Of the studies included in the review, none provided information relating to the remaining implementation factors proposed in Durlak’s framework.

### Summary of Evidence and Conceptual Model

**Fig. 2:**
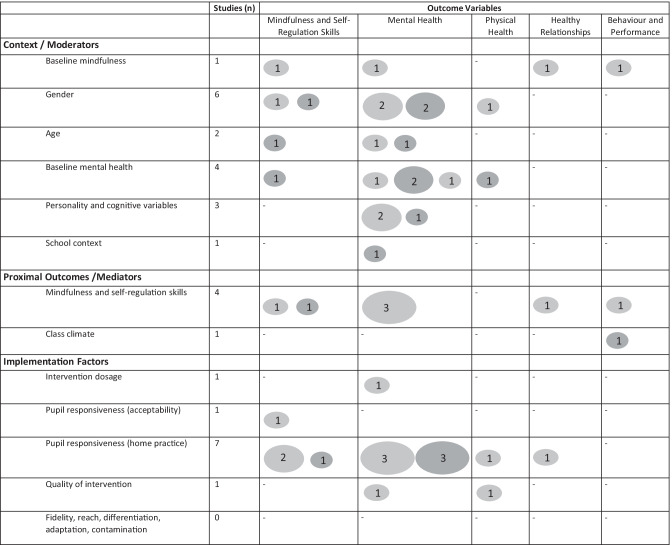
Evidence gap map for SBMT split by moderators, mediators, and implementation factors. The total number of non-significant findings (red circle), significant findings (green circle) and opposite direction findings (yellow) identified from the quantitative papers that evaluated moderators, mediators, and/or implementation factors. The size of the circle indicates amount of evidence, where a smaller circle indicates less evidence whereas a larger circle indicates more evidence. The number of studies (*n*) indicates how many included papers in the scoping review evaluated the specified variables (moderators, mediators, or implementation factors) in relation to the categorized outcomes. Note that there are generally more findings reported than number of papers given that papers tended to address multiple outcomes. Outcomes were categorized by five large outcome categories: mindfulness and self-regulation skills, mental health, physical health, healthy relationships with others and the physical world, and school behaviour and (academic) performance (Roeser et al., 2020). For our included papers, the outcomes categorized as mindfulness and self-regulation skills include emotional intelligence, emotional regulation, emotional awareness, clarity, impulse control, mind wandering, and affective self-regulatory efficacy. The outcomes categorized as mental health include depression, anxiety, weight/shape concerns, negative/positive affect, thin-ideal internalization, well-being, stress, positive attitudes, mood disturbance, psychosocial impairment, aggression, somatic complaints, and resilience. The outcomes categorized as physical health include adherence to physical activity, dietary restraint, and substance use. The outcomes categorized as healthy relationships with others and the physical world include empathy, classroom climate, relationships, and social connectedness. The outcomes categorized as school behaviour and (academic) performance include competence and academic motivation. For gender as a moderator, the significant findings were coded as findings where girls demonstrated greater benefits whereas the opposite direction findings were coded as findings where boys demonstrated greater benefits

**Fig. 3:**
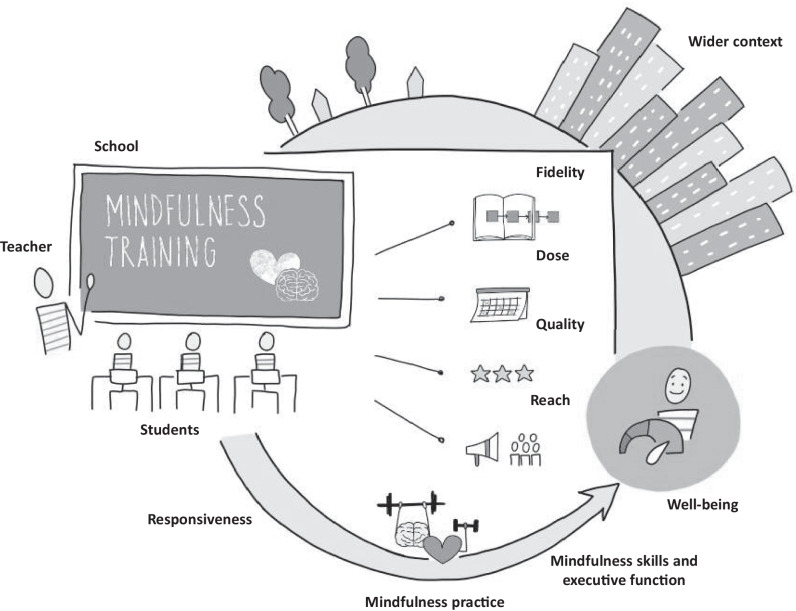
Conceptual model of SBMT including moderators, mediators, and implementation factors. The model proposes that the broader school context and the characteristics of the school community in which the SBMT is being implemented may moderate its effects on proximal and distal outcomes. Pupil baseline characteristics, including mental health and socio-demographic variables (e.g. age, gender, ethnicity), may also moderate the effect of SBMT on outcomes. Potential mediators include (**a**) executive function (as an umbrella term for a variety of self-regulation skills) and (**b**) levels of mindfulness skills learned during the training and enhanced through responsiveness and practice. It is also plausible that changes in operational features of the school might act as mediators of programme effectiveness. For example, programme implementation may change the overall classroom and school climate or teacher mental health, which then subsequently impacts individual pupil outcomes. The model also incorporates the potential moderating role of implementation factors known to influence SEL programmes more generally and potentially SBMT programmes as well (e.g. fidelity, dose, quality, and reach). Well-being is used here to represent outcomes assessed following implementation of SBMT (e.g. distal mindfulness skills and executive function, mental health, physical health, healthy relationships, or pupil behaviour and performance). More details related to the use and definitions of the theoretical model terms can be found in Online Resource 5. Design by Kim Haesen

Based on the findings of the scoping review, we present a summary of existing evidence and current gaps (Fig. 2), and a conceptual model (Fig. 3) to guide theory-led developments. Details related to the use of the theoretical model terms can be found in Online Resource 5. While research to date has included a range of pupil-related outcomes, we differentiate distal outcomes (e.g. mental health) from the proximal outcomes that are hypothesised as mediators (e.g. mindfulness skills, and executive function, which encompass many of the self-regulation skills taught in SBMT).

The model proposes that the broader school context and the characteristics of the school community in which the SBMT is being implemented may moderate its effect on proximal and distal outcomes (Ford et al., 2021). There is a growing literature on the importance of the school context in facilitating or frustrating the implementation of evidence-based programmes in schools. For example, the school context and community may influence adoption and fidelity by providing a supportive or restrictive implementation setting (Domitrovich et al., 2019; Nguyen et al., 2021). Future research should investigate different dimensions of school context and implementation to examine how they interact to create desired student- and school-level outcomes following SBMT. Pupil baseline characteristics, including mental health and socio-demographic variables (e.g. age, gender, ethnicity) may also moderate the effect of SBMT on outcomes. On the other hand, based on theory (Roeser et al., 2020) and the scoping review findings, potential mediators include executive function and mindfulness skills learned during the training and enhanced through practice, on the theoretical premise that mindfulness training aims to improve the self-regulatory components that these two general mental processes entail (Kuyken et al., 2013). It is also plausible that changes in operational features of the school might act as mediators of programme effectiveness (Jennings & Greenberg, 2009). For example, programme implementation may change the overall classroom and school climate or teacher mental health, which then subsequently impacts individual pupil outcomes. The model also incorporates the potential moderating role of implementation. In general, the scoping review found existing but limited evidence that pupil responsiveness, home-practice, dose, and quality of the mindfulness instructor may relate to SBMT effectiveness. This is consistent with a meta-analysis of school-based SEL programmes that highlighted implementation problems result in smaller effect sizes (Durlak et al., 2011).

The intention is that this SBMT conceptual model can serve as a framework to use when testing mediation, moderation, and the effects of implementation in the context of secondary school students. Based on the findings of the scoping review, some recommendations generated for future research include (1) using this conceptual model to inform trial design and process evaluations; (2) using appropriate statistical analyses to test for effects; and (3) considering possible implementation issues when interpreting results. Other recommendations also include incorporating consistent measures of potential moderators (e.g., comparable cut-offs for age), mediators, and outcomes to avoid heterogeneity, as well as clarifying programme components, such as specific mindfulness practices implemented. Furthermore, the model’s consideration of moderators, mediators, and implementation factors could also potentially be used to guide testing of mindfulness training in other populations and contexts; for example, in mindfulness training for younger children and for adults in workplaces or prisons, which are developing fields of research (Galante et al., 2021).

## Discussion

The objectives of this paper were to (1) elucidate the moderators, mediators, and implementation factors in universal SBMT; (2) scope the literature and summarise the findings on outcomes; and (3) provide a conceptual model for future SBMT research. In contrast to the broader SEL literature, this review highlights the scarcity and limitations of studies providing process evaluations of SBMT. Therefore, it is currently unclear for whom they work best, the processes through which effects are exerted, and in what context they are most effective or ineffective. We have developed a summary of existing evidence relating to SBMT for adolescents, identifying gaps, and propose recommendations for future research, grounded in a novel model for SBMT.

### Moderation: Reconceptualising Effectiveness

Gender has been the most studied potential moderator, with girls reporting greater benefits on self-regulation and mental health (Butzer et al., 2017; Johnson et al., 2016; Kang et al., 2018), while boys report greater benefits in physical health (Salmoirago-Blotcher et al., 2018). However, no gender-related effects on mental health and mindfulness have been observed (Johnson et al., 2017; Van der Gucht et al., 2017). Whilst one study showed that poor baseline mental health was related to benefits on mental health (Van der Gucht et al., 2017), other studies reported no benefits of this sub-group on mindfulness, mental health, and physical health (Atkinson & Wade, 2015; Johnson et al., 2017); and one study observed that mental health got worse for those with lower baseline levels of mental health (Johnson et al., 2016). We identified two studies that examined age, with older adolescents obtaining more improvements on mental health (Van der Gucht et al., 2017), but also no age effects have been observed (Johnson et al., 2017). High baseline mindfulness facilitated improvements in self-regulation, mental health, healthy relationships and behaviour, and academic performance (Lombas et al., 2019). In terms of personality, high attachment anxiety, as well as high agreeableness, and low emotional stability were related to greater improvements in mental health (Campbell, 2015; Huppert & Johnson, 2010), while cognitive factors showed no effects on mental health (Rice et al., 2015). Recent evidence indicates that the school context (e.g. urbanity), school community (e.g. school deprivation), and operational features of schools (e.g. school climate) explain a small but significant variation in students’ psychological outcomes (Ford et al., 2021). Only one study in the current review examined the influence of school factors on students following participation in a SBMT with no effects (Van der Gucht et al., 2017).

Universal school-based programmes may have various impacts: provide treatment for a diagnosable problem, prevent transitions into a diagnostic problem, and promote positive outcomes that may enhance adolescents’ academic performance and mental health (Greenberg & Abenavoli, 2017). However, there is evidence that policy making in education overlooks the more nuanced effects of universal prevention programmes. Traditional research of targeted programmes uses standard effect size statistics (e.g. Cohen’s d) to quantify change (Kraft, 2020). Effect sizes may under-represent the change for low frequency, yet important, outcomes. For trials of universal SBMT, the majority of participants have low levels of mental health problems and therefore little room for change on diagnostic measurement scales before and after a programme. A small proportion of participants will have an existing diagnosable condition and very large sample sizes are required to detect small changes in this subgroup. Thus, future trials of universal SBMT may better identify important changes by using outcomes of relative risk of developing adverse outcomes or relative odds of improved positive outcomes among relevant subgroups (Hansen, 2020). Moreover, within a school population, some sub-groups may experience benefits, and others may report deterioration, of differing degrees (Greenberg & Abenavoli, 2017).

### Mediation: Looking for the Pathways of Change

To understand the processes by which a SBMT exerts its effects, our scoping review reported on studies that incorporated mediation analyses. A total of five studies evaluated potential mediators of SBMT on student outcomes. There is evidence that improvements in mindfulness are associated with improvements in mental health (Britton et al., 2014), and specifically that mindfulness and cognitive reactivity might mediate the effect of SBMT on mental health, healthy relationships, and performance (Lombas et al., 2019; Van der Gucht et al., 2018). However, no mediational effects of mindfulness and self-compassion on self-regulation (Daly et al., 2015), and of classroom climate on behaviour and performance (López-González et al., 2018) were observed. Most studies testing mediation do not study change in the mediator prior to the change in the outcome, typically including only two time points; this means findings cannot speak to mechanisms of change. Thus, we recommend study designs with at least three time-points to consider temporal precedence (change in mediation predicting later change in outcome). The field of analysing mediators of SBMT is still in its infancy. Our model suggests specific variables with some empirical basis and theoretical foundation, such as mindfulness skills and executive function.

### Implementation: Universal SBMT in Practice

We know that implementing universal SEL and mental health prevention programmes with care to important implementation dimensions has a significant impact on effectiveness (Durlak & DuPre, 2008; Durlak et al., 2011). Our review suggests that evidence is limited in studies of SBMT; very few studies statistically examined how programme implementation related to outcomes. Participant responsiveness was the most commonly measured factor; however, operationalization varied across studies. It is important to operationally define indicators of responsiveness and how they might relate to outcomes. For example, one could benefit from a programme despite finding it unenjoyable (e.g. a filling at the dentist). Generally, mean responsiveness fell in the middle of scales but self-reported home practice was low. Limited information about distribution of responsiveness means subgroup analyses cannot be used to assess the relative benefits or harm for those who respond positively compared to those who responded poorly. Nevertheless, it was demonstrated that higher responsiveness (e.g. satisfaction) with the SBMT programme might be associated with improvements in self-regulation (Metz et al., 2013). Although most studies reported low levels of student mindfulness practice, it has been observed that the level of practice could predict mindfulness and self-regulation, mental health, physical health, and healthy relationships (Broderick & Frank, 2014; Frank et al., 2021; Huppert & Johnson, 2010; Kuyken et al., 2013), but other studies found no effects of practice on self-regulation and mental health (Huppert & Johnson, 2010; Johnson et al., 2016, 2017; Lam & Seiden, 2020). One study showed that higher SBMT dose was related to increases in mental health and positive attitudes towards school (Khalsa et al., 2012). It was also observed that an optimally trained facilitator (e.g. high quality of delivery) might lead to greater effects on mental health and physical health (Atkinson & Wade, 2015). Some studies measured fidelity, but no studies examined the influence of fidelity on outcomes. Moreover, no studies commented on the reach of SBMT within a school.

Durlak and DuPre (2008) define programme reach as “the rate of involvement and representativeness of intervention participants”, and together with fidelity, dose, and quality, configures the group of implementation factors that have been studied more in promotion and prevention programmes in different community settings. For studies where SBMT reaches every pupil in the school, we hypothesise that this may influence a change in the whole school culture compared with studies that deliver the programme to one or two classes. However, as we have mentioned above, no studies report on the proportion of students receiving SBMT relative to the whole school and, therefore, we cannot draw any conclusions about the influence of programme reach. All the included studies examined classroom-based programmes (i.e. “stand alone” interventions delivered over a period of weeks). Evidence from the SEL literature highlights the importance of broadening the classroom approach to school-wide, where the unit of change is the whole school community and aims to integrate SEL into daily interactions at multiple settings (Jones & Bouffard, 2012; Meyers et al., 2019; Oberle et al., 2016). Future SBMT should consider this approach to examine whether long-term and sustainable school-wide implementation of daily mindfulness interactions with all students and staff results in more positive outcomes. The conceptual model proposed here outlines how future studies can incorporate analyses relating to implementation.

### Recommendations for Future Research

The majority of included studies did not report whether the moderation, mediation, or implementation analyses were pre-specified. Thus, it cannot be determined whether findings reflected only the variables tested or whether many more were tested with null effects and were not reported. A meta-analysis of SBMT for children and adolescents highlighted evidence of publication bias (Dunning et al., 2019), and it is possible this is also the case for exploratory moderation, mediation, and implementation analyses reported here. Future research should incorporate pre-published trial protocols and statistical analysis plans. Researchers should also be encouraged to be transparent about for whom SBMT may be ineffective or even harmful. There is limited evidence for adverse effects and potential harm in the context of mindfulness-based programmes in adult populations (Baer et al., 2019, 2021) and, to our knowledge, no evidence of adverse effects or potential harm in the context of universal SBMT for adolescents. However, this is likely due to under-reporting, and it should become routine practice in the same way that it is for pharmacotherapy trials.

A lack of long-term follow-ups or consideration of the sustainability of SBMT was evident in our review. The challenges of achieving long-term follow-up of programmes and collection of long-term data in school-based studies are well documented (Dray et al., 2017; Ellickson et al., 1988). While long-term studies are difficult to conduct, evidence shows that some universal school-based programme trials for mental health have delayed effects that are only identifiable in later time points (Calear & Christensen, 2010). Therefore, longer-term follow-ups are recommended.

A related issue is statistical power to detect moderators, mediators, and implementation factors. None of the studies included in the current review was adequately powered to test for these effects; and thus, seeking significant vs non-significant findings can be misleading. Sample sizes large enough for such analyses are difficult to fund and implement, making it a challenge to test for these important effects. If study sample size does not allow for such analyses, exploratory analyses are nonetheless useful, e.g. presenting the characteristics of pupils who report particularly high or low scores on responsiveness scales and mindfulness practice, and whether this relates to subsequent outcomes, would identify those for whom SBMT is suitable and unsuitable. Reviews and meta-analyses can draw on these and report on pooled effects.

Protocols of future trials should clarify what elements of mindfulness are included in the training. Out of the included studies, there were many that indicated that they were using Mindfulness-Based Stress Reduction (MBSR) or Mindfulness-Based Cognitive Therapy (MBCT) adaptations. However, typically limited information was provided on which elements of MBSR/MBCT (e.g. types of practices) were used. Future studies should provide more information on the types of programmes to assess which components are most effective for adolescents. Programmes should then be standardised to include these core elements so that they can be directly comparable.

Finally, it is essential that future research considers not only the cost-effectiveness of distinct SBMT training routes in terms of e.g. intensity (Crane et al., 2020), but also the opportunity cost should it shift resources away from other activities, which could move SBMT towards a different cost-effectiveness position.

### Strengths and Limitations

Strengths of the current review cover the inclusion of a broad range of outcomes, the pooling of evidence by outcomes, and the adoption of an open and comprehensive approach by locating as many studies as possible and including grey literature (e.g. dissertations and theses). On the contrary, limitations include the fact that our search strategy identified papers that evaluated mediation, moderation, or implementation, and it is therefore possible that some papers were excluded if they did not state this explicitly. Similarly, due to inconsistent definitions of implementation, it is possible that some papers were excluded if they applied definitions outside of the scope of our search strategy. A proportion of articles reported that mediation was tested, but in general the methods did not meet the statistical requirements to do so (Kazdin, 2007). While such studies are not adequately testing for effects, they were included to present the relatively small field of SBMT as it stands now. All in all, we propose a conceptual framework that functions as a summary balancing previous research that has some, albeit limited, empirical evidence and new potential avenues for the field. Thus, we have synthetized those aspects that have received more consideration. However, this does not mean that other factors (e.g. implementation factors such as adaptation, monitoring of control group) do not merit future research.

## Conclusions

SBMT has the potential to be delivered universally to improve mental health and well-being. Several programmes have been developed in the past two decades. However, implementation of SBMT has outpaced research on its potential effectiveness across diverse school contexts and pupil characteristics. This scoping review suggests that the field is still in its infancy with regard to understanding the impacts of SBMT, the processes through which SBMT exerts its effects, and the influence of implementation factors on outcomes. Our review suggests that gender, mindfulness and self-regulation, and student mindfulness practice, were the most studied moderators, mediators, and implementation factors, respectively, and mental health the most studied outcome. Other potential moderators, mediators, implementation factors, and outcomes need further research (Fig. 2). As the use of SBMT for adolescents continues to grow, more evidence is required relating to their differential effects across students and school contexts, alongside their pathways of change, and the relative importance of implementation quality. We offer a conceptual model (Fig. 3) and specific recommendations for future research.

## Supplementary Information

Below is the link to the electronic supplementary material.

Supplementary file1 (DOCX 64 KB)

Supplementary file2 (DOCX 106 KB)
