# Supplementary material for: Universal Mindfulness Training in Schools for Adolescents: a Scoping Review and Conceptual Model of Moderators, Mediators, and Implementation Factors
Source: Prev Sci. 2022 Mar 10;23(6):934–53. doi: 10.1007/s11121-022-01361-9 (PMC9343282; doi:10.1007/s11121-022-01361-9)
Supplement: Supplementary file 1 — Supplementary file1 (DOCX 64 KB) [file 11121_2022_1361_MOESM1_ESM.docx]

**Online Resource 1** Search Query

Search: **((((((((((mindful*) OR (Mindfulness[MeSH Terms])) OR (mindfulness-based)) OR (MBI)) OR (MBP)) OR (meditation*)) OR ("school-based mindfulness")) OR ("contemplative education")) AND ((((((((((((((((((((((((mediat*) OR (moderat*)) OR (mechanism*)) OR ("mechanism of change")) OR ("mechanisms of change")) OR (implement*)) OR (engagement)) OR (fidelity)) OR (dose)) OR (dosage)) OR (feasibility)) OR (acceptability)) OR (program evaluation)) OR (programme evaluation)) OR (context)) OR (facilitation)) OR ("program reach")) OR ("programme reach")) OR ("participant reach")) OR (adaptation)) OR (responsiv*)) OR ("program differentiation")) OR ("programme differentiation")) OR ("monitoring of control conditions"))) AND (((((((((((((((((((((((((((((((school*) OR (schools[MeSH Terms])) OR (school-based)) OR (classroom*)) OR (classroom-based)) OR ("high school")) OR (college*)) OR (campus*)) OR ("elementary school")) OR ("middle school")) OR ("multicultural school")) OR ("private school")) OR ("public school")) OR ("primary school")) OR ("secondary school")) OR ("boarding school")) OR ("charter school")) OR ("institutional school")) OR ("junior high")) OR ("school environment")) OR ("special school")) OR ("elementary education")) OR ("multicultural education")) OR ("private education")) OR ("public education")) OR ("primary education")) OR ("secondary education")) OR ("institutional education")) OR ("special education")) OR ("non-traditional education")) OR ("academic settings"))) AND ((((((((((((((((((((((((Students[MeSH Terms]) OR (student*)) OR (adolesc*)) OR (adolescent[MeSH Terms])) OR (youth)) OR ("young people")) OR ("young adult")) OR (child*)) OR (kids)) OR (juvenile*)) OR (pupil*)) OR (kid)) OR (teen*)) OR (preadolesc*)) OR (puberty)) OR (pubescen*)) OR (prepube*)) OR (late childhood)) OR (teach*)) OR (educator)) OR (school-teacher)) NOT ("medical students")) NOT ("nursing students")) NOT ("university students"))**

**Online Resource 2** Additional details of included studies

| ***Author***  ***(year)*** | ***Country*** | ***Class-wide implementation (yes-which classes / no)*** | ***Online delivery during school hours*** | ***Minutes x session*** | ***Sessions x week*** | ***Nº weeks*** | ***Who implemented*** | ***Manualised training*** |
| --- | --- | --- | --- | --- | --- | --- | --- | --- |
| Anand (2012) | India | Yes - The intervention was delivered during library period | No | 40 | 1 | 8 | NR | Yes - The intervention was based on MBSR and the stress reduction workbook for teens by Biegel (2009). |
| Atkinson (2015) | Australia | No - Grade-level  (Grades 10-12) | No | NR | 1 | 3 | External - Postgraduate psychology students | Yes - Some mindfulness exercises were adapted from MBCT for Depression |
| Bauer  (2020) | USA | Yes - The intervention was delivered during the last class period for sixth graders | No | 45 | 4 | 8 | External - Individuals who had practical knowledge and experience in mindfulness | Yes - The intervention was adapted by 'Calmer Choice' (n.d.) |
| Bergen-Cico (2015) | USA | Yes - The intervention was delivered during English Language Arts Courses | No | 4 | 3 | ~ 40 | Internal - A certified public-school teacher who had completed 30-hour training in the 'YogaKids' programme, a 200-hour registered yoga teacher training through 'Yoga Alliance,' and had a personal mindfulness practice | Yes - The intervention was inspired by the 'YogaKids' programme which adapts hatha yoga for children |
| Britton (2014) | USA | Yes - The intervention was delivered during the History class period | No | 3 to 12 | 1 | 6 | Internal - Two history teachers: 1 who completed Roth's Integrative Contemplative Pedagogy (ICP) training and had 5 years of mindfulness experience and 1 with no prior mindfulness experience but had completed MBSR | Yes - The intervention was based on Roth's Integrative Contemplative Pedagogy (ICP) curriculum |
| Broderick (2014) | USA | Yes - The intervention was delivered as a replacement for the DARE curriculum | No | NR | NR | NR | Internal - Guidance counselors and the assistant principal facilitated the sessions and teachers met weekly to review activities | NR |
| Butzer (2017) | USA | Yes - The intervention was delivered during the PE class period | No | 35 | 1 to 2 | ~26 | External -Two teachers and five assistants who were not members of the school staff. All teaching staff were certified as 200h hour yoga teachers and 4/5 assistants completed the Kripalu Yoga in Schools (KYIS) curriculum. | Yes - The intevention was adapted from the 'Kripalu Yoga in Schools (KYIS)' curriculum (KYIS, 2015) |
| Campbell (2015) | USA | Yes - The intervention was delivered during the English class period | No | NR | 1 | 6 | Both - Staff and volunteers taught the curriculum and had various levels of mindfulness experience | Yes - The intervention was based on the ‘.b’ ('Dot Be') curriculum |
| Chancey (2018) | USA | Yes - The intervention was delivered during health and PE class periods | No | 30 | 2 | 6 | External - Doctoral students | Yes - The intervention was based on the 'Learning to BREATHE' curriculum |
| Clarke (2021) | USA | Yes - The intervention was delivered during PE or dance class periods | No | 60 | 1 | 6 | External - Project staff consisting of a certified yoga instrucor/social worker and a master's level clinician trained in mindfulness/counseling | NR |
| Daly  (2015) | USA | Yes - The intervention was delivered during PE class periods | No | 40 | 3 | 16 | External - 'Bent on Learning' teachers facilitated the sessions adnd were required to have a 200-hour yoga certification and two years of teaching experience | Yes - The intervention was based on the 'Bent on Learning' teacher training manual |
| Frank  (2021) | USA | Yes - The intervention was delivered during health education class periods | No | NR | NR | 12 | Internal - Teachers were trained by the programme developer and an on-site coach for four weekly training sessions (6 hours in total) and then teachers attended a 2-day training on implementing the curriculum | Yes - The intervention was based on the 'Learning to BREATHE' curriculum |
| Huppert (2010) | UK | Yes - The intervention was delivered during religious studies lessons | No | 40 | 1 | 4 | NR | Yes - The intervention was based on MBSR |
| Johnson (2016) | Australia | Yes - The intervention was delivered during pastoral care, community projects, or English, Science, or History class periods | No | 35 to 60 | 1 | 8 to 9 | External - A mindfulness practioner with ten years of personal mindfulness practice who had undergone ‘.b’ and adult facilitator training | Yes - The intervention was based on the ‘.b’ ('Dot Be') curriculum |
| Johnson (2017) | Australia | Yes - The intervention was delivered during pastoral care, community projects, or English, Science, or History class periods | No | 35 to 60 | 1 | 8 to 9 | External - A mindfulness practioner with ten years of personal mindfulness practice who had undergone ‘.b’ and adult facilitator training | Yes - The intervention was based on the ‘.b’ ('Dot Be') curriculum |
| Johnson (2019) | Australia | Yes - The intervention was delivered during English, Christian studies, health/PE, or extended home group class periods | No | 90 | 1 | 8 | External - A mindfulness practioner with ten years of personal mindfulness practice who had undergone ‘.b’ and adult facilitator training | Yes - 'Mindfulness Training for Teens (Dewulf, 2013) which is baed on MBSR and MBCT |
| Kang  (2018) | USA | Yes - The intervention was delivered during History class periods | No | 3 to 12 | 4 to 5 | 6 | Internal - Two history teachers: 1 who completed Roth's Integrative Contemplative Pedagogy (ICP) training and had 5 years of mindfulness experience and 1 with no prior mindfulness experience but had completed MBSR | Yes - The intervention was based on Roth's Integrative Contemplative Pedagogy (ICP) curriculum |
| Khalsa (2012) | USA | Yes – (NR) | No | 30 to 40 | 2 to 3 | 11 | External - Yoga Ed teachers who had undergone 200-hour yoga teacher training in Kripalu yoga | Yes - The intervention was adapted by the 'Yoga Ed' programe for secondary schools (http://www.yogaed.com) |
| Kuyken (2013) | UK | Yes - The intervention was delivered during religious studies, or personal, social, and health education class periods | No | NR | 1 | 9 | Internal - Teachers trained in the MiSP (Mindfulness in Schools Programme) curriculum or who had expressed an interest to be trained | Yes - The intervention followed the MiSP (Mindfulness in Schools Programme) curriculum which is based on MBCT and MBSR |
| Lam  (2020) | China | Yes - The intervention was delivered during religious or social studies lessons | No | 70 | ~ 1/month | 6 | External - Clinical and school psychologist who had MBCT/MBSR training | Yes - The intervention was based on the 'Learning to BREATHE' curriculum |
| Lawson (2019) | USA | Yes - The intervention was delivered during English lessons | No | 2 or more | NR | NR | Internal - The classroom teacher was trained on breathing exercises by the researcher and through training exercises through the Mind Body Awareness Project (2017) | Yes- The exercises were based on the Mind Body Awareness Project (2017) |
| Lombas (2019) | Spain | Yes – (NR) | No | 5 | 2 | 18 | Internal - Teachers facilitated the mindfulness sessions and were trained by the authors for a total of 16 hours | Yes - The intervention followed the Happy Classrooms Programme (HCP; Arguis et al, 2012) |
| López-González (2018) | Spain | NR | No | NR | NR | NR | External - Certified trainers of the University of Barcelona | NR - The intervention was conducted during TREVA's Relaxation-Mindfulness Program (López-González, 2013; López-González, Álvarez, & Bisquerra, 2016) |
| Metz  (2013) | USA | Yes - The intervention was delivered during concert choir course elective classes | No | 15 to 25 | 1 | 18 | Internal - The choir director taught the Learning to BREATHE programme and attended an 8-week MBSR programme and completed a 2-day in-service training for Learning to BREATHE | Yes - The intervention was based on the 'Learning to BREATHE' curriculum |
| Mrazek (2019) | USA | Yes - The intervention was delivered during graphic production, entrepreneurism, computer science, AP physics, AP psychology, or special education classes | Yes | 4 to 12 | NR | 22 | External - The intervention was a 22-day digital course that could be accessed by students' computers, tablets, or phones. Teachers were encouraged to have students complete the lessons during class. | NR |
| Rice  (2015) | UK | Yes - The intervention was delivered during personal health and social education (PHSE) classes | No | 50 | 1 | 8 | External - Educational psychologists | Yes - Mindfulness-based CBT |
| Salmoirago-Blotcher  (2018) | USA | Yes - The intervention was delivered during health education class periods | No | 45 | 1 | 8 | External - Certified mindfulness teacher | Yes - The intervention was based on MBSR |
| Sibinga (2016) | USA | Yes - The intervention was delivered during 'resource' class time | No | NR | NR | 12 | External - Two experienced MBSR instructors both with personal mindfulness experience and > 10 years' experience teachin mindfulness | Yes - The intervention was based on MBSR |
| Van der Gucht (2017) | Belgium | Yes – (NR) | No | 100 | 1 | 8 | External - Certified mindfulness teacher | Yes - The intervention was based on MBSR and MBCT |
| Van der Gucht (2018) | Belgium | Yes – (NR) | No | 100 | 1 | 8 | External - Certified mindfulness teacher | Yes - The intervention was based on MBSR and MBCT |
| Worthen (2019) | USA | Yes - Mindfulness education program was a course requirement | No | 40 | 1 | 10 | External - Author of paper / mindfulness instructor | Yes - The intervention was based on Mindfulness-Based Cognitive Therapy 'Finding Peace in a Frantic World' (Williams & Penman, 2011) |

NT: not reported

**Online Resource 3.** Moderators and mediators of SBMT

| **Author (year)** | **Moderating / Mediating variable(s)** | **Outcome measure(s)** | **Time points** | **Method** | **Findings** |
| --- | --- | --- | --- | --- | --- |
| Atkinson (2015) | **Moderator:** Baseline mental health *(high risk versus low risk)* | Weight and Shape Concern (EDE-Q), Negative Affect (PANAS-X), Dietary Restrain (DEBQ-R), Thin-Ideal Internalization and Socio-Cultural Pressures (SATAQ-3), Eating Disorder Symptoms (EDE-Q), Psychological Impairment (CIA). | 4 (pre, post, 1 month post, 6 months post) | **Linear mixed models.** Baseline observations were used as covariates, resulting in a condition (mindfulness, dissonance, control) x time (post, 1-month, 6-month) x risk (low, high) fixed effects model for each outcome variable, with random effects accounting for individual and school-level variation. | There was a marginally signiﬁcant simple effect of condition among low risk participants [F (2,308.72) 5 2.90, p 5 .056], with pairwise comparisons showing those receiving the dissonance condition to be slightly lower on negative affect than control participants (p 5 .050; d=0.31 [0.05, 0.57]). There was no signiﬁcant effect of condition among high risk participants [F (2,313.56) 5 1.01, p 5 .365]. No effects were observed in the mindfulness condition. |
| Britton (2014) | **Mediator:** mindfulness (CAMS-R). | Behavioural and emotional problems (YSR), anxiety (STAI). | 2 (pre, post) | **Pearson product–moment correlation coefficients** were used to assess whether changes in mindfulness were associated with improvements in affect and clinical syndrome. | Improvements in CAMS-R total score among meditators were significantly correlated with reductions in overall affect disturbance (r = −.44, p = .001) and with increases in anxiety (r = .43, p = .002) as measured by the STAI. |
| Butzer (2017) | **Moderator:** Gender *(male versus female)* | Mood (BRUMS). Perceived stress (PSS). Impulsive behaviour (IBS). Emotional self-control (KWSCS). Affective Lability (ALS). Risk Behaviour (YRBS-MS). | 4 (1 week pre, 1 week post, 6 months post,1 year post) | **Split-plot analyses of variance (ANOVAs).** Condition (intervention; control) and gender (male; female) as the between-subjects factors, and time (baseline; end-program; follow-up 1; follow-up 2). Gender was included as a between subjects factor post-hoc. | Post hoc analyses conducted separately for males and females in each group intervention; control revealed that females in the intervention group reported statistically signiﬁcant increases in emotional self-control between pre-intervention versus 1 year follow up, 1 week follow up versus 1 year follow up, and 6 months follow up versus 1 year follow up, whereas males in the intervention group did not report signiﬁcant changes in emotional self-control over time. Conversely, males in the control group reported statistically signiﬁcant increases in emotional self-control between time 1 (1 week pre intervention) versus time 4 (1 year post), time 2 (1 week post) versus time 4, and time 3 (6 months post) versus time 4, whereas females in the control group did not report signiﬁcant changes in emotional self-control over time. |
| Campbell (2015) | **Moderator:** Attachment anxiety *(high versus low)* | Perceived stress (PSS). Attachment style (ECR-R-GSF), emotion regulation (DERS), positive and negative affect (PANAS-SF) | 2 (1 week pre, 1 week post) | **Step-wise regressions.** A four-level step-wise model was created, with the first level including dummy-coded  covariates for age, race/ethnicity, gender, and participation in the previous MM mini-course, the second level also including the independent variable, group, the third level adding the centered moderator variable, and the fourth level also including the dummy-coded interaction variable. | The model including the interaction term accounted for a significantly greater amount of variance than the model without for negative affect only, with F change (1, 910) = 7.427, R2 change = .01, p = .007. This result was supported by a review of the individual predictors, with results showing that anxious attachment was a statistically significant moderator for negative affect, with t(909) = -2.707, p = .007, sr2 = .01. |
| Daly (2015) | **Mediator:** Mindful awareness. (MAASA). Self-compassion (SCS). | Emotion regulation (ERICA and ERC), Mindful awareness (MAASA). Interoceptive Awareness (MAIA). | 2 (pre, post (>2 weeks)) | Change scores from PRE to POST intervention, were calculated for each of the potential mediating variable scales. Correlation analyses were run with change scores for emotion regulation. | Mindful attention and self-compassion were not significantly correlated with increases in emotion regulation. Body awareness (MAIA) change scores were significantly, positively correlated with emotion regulation (ERICA) change scores. Due to the non-significant findings between mindful awareness and self-compassion with emotion regulation, mediation analyses were not conducted. |
| Huppert (2010) | **Moderator:** Personality *(agreeableness and emotional stability high versus low)* | Mindfulness (CAMS-R), resilience (ERS), well-being (WEMWBS), personality (TIPI) | 2 (1 week pre, 1 week post) | **Multiple regressions.** To assess the effect of condition (mindfulness training or control) and personality on the standardised residual outcome measures (mindfulness, resilience and well-being). | The change in well-being was associated with several of the baseline personality measures: agreeableness (β = 0.324, p = 50.01); emotional stability (β = 0.243, p = 50.05); and openness to experience (marginal, β = 0.194, p = 50.10). |
| Johnson (2016) | **Moderator:** Gender *(male vs female).* Baseline depression and anxiety using DASS-21 *(high vs low).* Weight concerns using Weight/shape subscales of the Eating Disorder Examination-Questionnaire (h*igh vs low) “For depression and anxiety, “high” classifications were based on scoring moderate or above (7 or 6 respectively)”* | Depression Anxiety and stress (DASS-21), Eating disorder examination (EDE-Q), Mental well-being (WEMWBS), Mindfulness (CAMM), Emotional regulation (DERS), Self-compassion (SCS). | 3 (One week pre- and post, 11 weeks post) | **Linear Mixed Model.** Given the absence of significant differences, LMM was used to investigate gender, depression, anxiety, and weight shape concerns as moderators. | All significant moderator by group by time interactions were found at three-month follow-up, where anxiety was higher in the mindfulness group compared to controls for males, and also for those with low baseline weight/shape concerns or low baseline depression. A subsequent independent t-test demonstrated that in our sample, males (M = 2.63, SD = .76) were higher at baseline on mindfulness than females (M = 2.23, SD = .73), t(272) = 4.36, p < .001, d = .54. |
| Johnson (2017) | **Moderator:** Age. Gender *(male vs female).* Socioeconomic status (SES). Baseline depression and anxiety *(high/low).* Weight concerns (h*igh/ low).* Weight and shape concern: *(subscales from the EDEQ)* | Negative affect: Depression Anxiety and stress (DASS-21), Weight and shape concern (subscales from EDE-Q), Mental well-being (WEMWBS), Mindfulness (CHIME-A). | 4 (3–4 weeks pre, post and 6- and 12-month post) | **Linear Mixed Model.** | There were no moderator-group-time interactions for any of the analyses. |
| Kang (2018) | **Moderator:** Gender | Anxiety (STAI-C), Cognitive affective mindfulness (CAMS-R), Self-compassion (SCS). | 2 (pre and post) | **Two Analyses of Variance (ANOVA)** for repeated measures using group (mindfulness vs. control) and gender (male, female) as two between factors and global affect disturbance and positive affect across the within-factor (pre-test vs. post-test) each as an outcome variable. | Gender effects in response to interventions. We tested whether there was a group (mindfulness, control) × time (pre-test, post-test) × gender (male, female) interaction with greater affective improvement due to mindfulness training among females compared to their male counterparts. Non-significant three-way interactions were detected for global affect disturbance, F (1, 96) = 0.70, p = 0.40, d = 0.17, and positive affect with a small to medium effect size, F (1, 96) = 1.83, p = 0.18, d = 0.29. |
| Lombas (2019) | **Moderator:** Baseline mindfulness. **Mediator:** Improvement in mindfulness. | Mindfulness (MAAS), Self-esteem (RSE), Life satisfaction (SWLS), Depressive symptoms (Scale of depressive symptomatology), Perceived stress (PSS), Basic psychological needs (ESNPE), Mood (TMMS), Aggression (School aggression scale), Classroom environment (Classroom environment scale), Empathy (ICEA), Academic motivation (28 items). | 2 (pre, 6 months post) | **Moderation: Multiple linear regression analysis,** with post-test mindfulness as an outcome variable, and group (0 for control group and 1 for intervention group), pre-test mindfulness and their interaction as predictor variables. In order to control for demographic characteristics, age and gender were also introduced as predictor variables. **Mediation: Longitudinal mediation analyses** were performed to test the mediation role of mindfulness between the intervention and the outcomes (Sobel, 1982). The bias-corrected bootstrap method (n=2000 resamples) was used to compute a 95% confidence. | Results showed that all predictors were significant. The interaction between group and pre-test mindfulness indicated that the intervention only had effect at medium and high levels of pre-test mindfulness. |
| Lopez- Gonzalez (2018) | **Mediator:** Class climate. | Mindfulness skills and relaxation (EHERMA), Classroom climate (EBCC).  Academic performance | 2 (pre and post) | The macro PROCESS of SPSS (Hayes, 2013) in order to analyse the mediating effect of CC in the relation to intervention arm. The nonparametric bootstrapping procedure was used with 5000 repetitions to calculate the confidence intervals of 95% of the indirect effects. | There was a significant effect of class climate on the relationship between the intervention and academic performance. The total effect was significant while the direct effect was not significant. |
| Rice (2015) | **Moderator:** Baseline cognitive variables (Reward seeking, negative self-beliefs, Over general memory) | Depressive symptoms (mood and feelings questionnaire), Reward processing (CGT), Negative self-beliefs (DASC), Autobiographical memory (SCEPT). | 2 (pre and 9 weeks post) | Tested whether cognitive variables at baseline moderated depressive symptom change by including an interaction term between the baseline cognitive variable and condition in the regression models | Reward-seeking at baseline did not moderate depressive symptom change in the MBCT group Negative self-beliefs did not moderate change in depressive symptoms in any of the three intervention groups. Over general autobiographical memory did not moderate any of the three intervention groups. |
| Salmoirago-Blotcher (2018) | **Moderator:** Gender, baseline physical activity (via accelerometers) | Feasibility (recruitment, retention, adherence). Moderate-vigorous physical activity (PAR), Dietary intake (Intake of fruit, vegetables, sodium, fish, and sugar-sweetened beverages), BMI. | 2 (pre and 6 months post) | **Quantile regression.** | Exploratory analyses by gender suggested that among males, median moderate-vigorous physical activity for the mindfulness intervention was higher at follow up compared to controls, particularly among those with higher levels of baseline physical activity. These associations were maintained 6 months, with a difference in median MVPA of 99 min/week in favour of HE-MT. |
| Van der Gucht (2017) | **Moderator:** Severity of depressive symptoms at baseline. Gender. Age NB no info to suggest not continuous. 'School track' (vocational vs technical vs general education) | Depression Anxiety and Stress (DASS-21). | 3 (pre, 1-week and 6 months post) | **Multi-level modelling.** Adding potential moderators and their interactions with condition and time to the condition model. | One significant and one marginally significant three-way interaction were found for the intervention group compared to the control group. A significant interaction effect was found for age at T2 (1 week post intervention), suggesting greater decrease in depressive symptoms for older students. This interaction effect is mainly however due to the difference in symptom scores in the control group, where students older than 15 showed an increase in symptoms at T2. A marginally significant negative interaction effect was found for baseline depressive symptoms at follow-up (NB time-point unclear in paper, this is the exact wording), suggesting greater decrease in symptoms during the 6-month follow-up for students with high levels of depression at baseline. At T2, the differences in declining trend between participants with high and low depressive symptoms at baseline can be explained only by accumulation of the main effects of condition (true treatment effect) and time (natural recovery or regression to the mean). No significant interaction effects were found for gender or school track. |
| Van der Gucht (2018) | **Mediator:** Cognitive reactivity and self-coldness | Depression Anxiety and Stress (DASS-21). | 3 (pre, 1-week and 6 months post) | The lower level time-lagged mediation model on the treatment group was estimated. Central to the analyses was that the proposed mediators served as lagged time-varying predictors of subsequent changes in the outcome. (Bauer et al., 2006). Random effects for both intercepts and coefficients were retained (Bauer et al., 2006). A model on the whole sample was re-estimated (including treatment and control group, dummy coded) for moderated mediation by including condition as a level-2 predictor in the model. Thus, moderated mediation was tested by examining if each potential mediator mediated the effect of time on outcome, and if the mediation effect was moderated by condition. | The indirect effect was significant for cognitive reactivity - decrease in cognitive reactivity post-intervention mediated the reduction of symptoms of depression, anxiety and stress. The indirect effect was also significant for self-coldness (a decrease in self-critical thinking mediated the effect on depression, anxiety and stress). Model coefficients indicate that MBI had a stronger effect on cognitive reactivity than the control condition, however, these differences was not significant. For depression and anxiety there was a statistically significant difference on the b-paths (i.e. the mediator to outcome relation) as a function of condition (the mediator was more strongly related to depression and anxiety in the MBI condition compared to the control condition). There was no statistically significant difference on the b paths as a function of condition for the outcome stress. For the model with self-coldness as the mediator, the cross-level interaction term in the model that tested for difference in a-paths (i.e. effect of time on the mediator at T2) as a function of condition (MBI versus control) was marginally significant, indicating that MBI had a stronger effect on self-coldness than the control condition. There was no statistically significant difference on the b-paths as a function of condition for the outcomes depression, anxiety and stress. |

**Online Resource 4.** Operationalisation of implementation factors and corresponding findings for studies reporting a measure of implementation

| **Author (year)** | **Implementation factor and operationalisation** | **Findings** |
| --- | --- | --- |
| Anand (2012) | **Dosage:** session attendance, (total=8). Participants included in analysis if completed 5/8 sessions.  **Participant responsiveness:**  usefulness, satisfaction, relevance of content, applicability, self-reported home practice [no. of days, duration of practice). **Quality:**  rating of instructor involvement. | **Dosage:** Not reported.  **Participant responsiveness:** mean number of days of practice was 47/56 days. Mean duration of formal practice was 8.57 (out of 10) minutes. 81% found intervention, "extremely useful" and 19% useful to some extent. 82% reported benefitting a great deal from the program and 17% reported "to some extent, and 1% as "little useful". Over all 83% were "extremely satisfied" and the rest (17%) were "satisfied." 62% reported that sitting meditation was most useful. Mindful eating was reported as very useful by 8%. In addition, 72% reported enjoying the role plays. None reported any of the program components to be useless. Furthermore, when asked whether they would recommend their friends to participate in the program 77% said "definitely" and 23% said "maybe yes." **Quality:** Students rated the instructor 87% felt it was "excellent", 10% felt it was "good" and 3% rated it as "satisfactory." |
| Atkinson (2015) | **Participant responsiveness:** (5-point scale (1= not at all, 5 = very much) for improvement in body image, enjoyment, amount of attention paid, extent of homework completed, understanding of concepts, ease of use, effectiveness, likelihood of continued use, home practice (5-point scale, not at all - a lot). **Quality:** MT instructor confidence (5-point scale, 1=not at all, 5= very much). Sub group analysis of participants receiving intervention from more experienced facilitator. | **Participant responsiveness:** Whole sample: Improvement in body image (M= 3.15, SD = 0.73), enjoyment (M=2.39, SD = 0.95), attention paid (M=3.03, SD= 0.97), homework completion (M=1.68, 0.92), understanding (M=3.58, SD = 1.03), ease of use (M=3.14, SD = 1.08), effectiveness (M= 2.39, SD= 1.08), likelihood of continued use (M=1.94, SD=1.07). **Quality.** MT instructor confidence (M=3.89, SD =1.08). **Findings for participants receiving intervention from more experienced MT instructor:** Improvement in body image (M= 3.14, SD = 0.76), enjoyment (M=2.49, SD = 0.77), attention paid (M=3.14, SD= 0.96), homework completion (M=1.71, 0.92), understanding (M=3.71, SD = 0.97), ease of use (M=3.27, SD = 1.08), effectiveness (M= 2.37, SD= 1.04), likelihood of continued use (M=1.94, SD=1.01). |
| Bauer (2020) | **Participant responsiveness:** Questions asked students to assess (5-point scale) (a) their overall rating of the class, (b) the amount of work they had to do, (c) the degree of active participation, and (d) how much practical knowledge they learned. | **Participant responsiveness:** Overall rating of class (M =3.23 SD=1.23), amount of work they had to do (M=2.6, SD=1.12), the degree of active participation (M=3.1, SD=1.28), how much practical knowledge learned (M=2.6, SD=1.31). |
| Bergen-Cico (2015) | **Dosage:** Class time was measured via weekly logs that the teacher used to record the mindful yoga practices. **Participant responsiveness:** Students asked to provide written reﬂective comments on their experiences with the intervention: ‘‘Did you feel that having yoga/meditation as a part of the class each day was helpful to you? Why/why not?’’ The student evaluations were not anonymous; however, students were assured that the evaluation would not affect their grades as their ﬁnal grades had been submitted. | **Dosage:** Intervention (3x 4 minutes per week) was delivered on 100 out of a possible 150 schools days. **Participant responsiveness:** 60 students (83 % response rate) provided written feedback on the mindful yoga practice. Of those, 60 % reported that they found the yoga/ meditation practices helpful. 25% said it improved concentration. 40% did not find the intervention helpful. 10% said it took away from class time. 10% said they were disappointed as the intervention did not help them concentrate/ focus and 5% said that the intervention made had the opposite effect (i.e. more distracted). |
| Britton (2014) | **Participant responsiveness:** Journal entries: Following meditation session, students given 5 minutes to "write about your experiences with the meditation today". | **Participant responsiveness:** Students produced a total of 633 journal entries about their experience (M=12.2 entries per student). 94% reported engaging with practice on nearly every occasion, 6% rejected practice at least once. 13% reported practice was boring. 92% reported a perceived benefit of practice. 82% reported feeling more focussed. 88% reported feeling more calm after practicing. |
| Broderick (2014) | **Participant responsiveness:** satisfaction, home practice. | **Participant responsiveness:** 86.5% of program participants were satisfied or very satisfied with the program. Approximately half of all participants reported that the most important skill they had learned from the program was how to deal better with stressful thoughts and feelings. 64.6% of participants indicated practicing some mindfulness techniques outside of class during the length of the program. Post-hoc analyses were completed to test whether mean gain scores for all outcome measures were statistically changed by the amount of time the student practiced being mindful outside of the programme. Somatic complaints were reduced for those practicing mindfulness for four or more days a week in comparison to both those practicing less than this, and those practicing only during the class. |
| Butzer (2017) | **Dosage:** Class attendance. **Participant responsiveness:** 12-item Yoga Evaluation Questionnaire (YEQ) was administered to the intervention group immediately after the yoga program had ended. Sample items include ‘‘How much do you like regular gym class (without yoga)’’ and ‘‘How much do you like yoga class.’’ Students rated their enjoyment of yoga and gym class on a visual analogue scale ranging from 0 (‘‘not at all’’) to 100 (‘‘very much so’’). The YEQ also included an item asking how often students practiced yoga outside of school on average, which asked participants to select from a series of responses ranging from 1 (‘‘never’’) to 6 (‘‘4–7 days per week’’). | **Dosage:** Dosage requirements were met, with an average student attendance rate of 95.75% (range across class sections = 93.87–97.24 %) and with instructors teaching an average of 30.75 minutes per class section (range = 29–32) (occasionally yoga sessions were cancelled due to snow days, assemblies, etc.). **Participant responsiveness:** How much do you like yoga class? (M=44.45, SD = 32.46). Examination of the histograms for these questions suggests that a 20 students chose extreme values (i.e., values of ‘‘0’’), which may have skewed the means. Home practice: M = 2.22, SD= 1.35). |
| Chancey (2019) | **Dosage:** attendance. **Participant responsiveness:** lasting value of programme (yes/no/unsure), importance, usefulness, programme effects on school (yes/no), general effect on well-being (yes/no), recommend programme to others (yes/no), home practice. **Fidelity. T**eachers completed fidelity checklists. **Adaptation:** Forms were reviewed after implementation and a review of the forms with corrective teaching was provided to the teachers when inaccuracies were noted in form completion. | **Dosage:** Not reported. **Fidelity.** Overall, the curriculum was implemented with 96.64% fidelity. Weekly implementation of all components ranged from 91.67% (week 2) to 100% (week 5). **Participant responsiveness:**  Lasting Value of Programme (yes- 74%, no- 4%, unsure-22%), Importance of the program (very important- 4%, important - 41%, neutral- 44%, unimportant - 4%, very unimportant - 7%), Usefulness: Components commonly reported as very useful were mindful breathing practice (34.8%), mindful movement practice (21.3%), and loving kindness practice (21.3%). Components commonly reports as not useful were the body scan (31.9%), group discussions (30.4%), and practice audios (44.7%). Programme effect on school (yes-32%, no - 68%). General effect on well-being (yes - 48%, no - 52%). Recommended programme to others (yes -91%, no - 9%). **Home practice.** Many students in the intervention group responded they never practiced at home either formally (35.6%) with audio downloads or informally (31.9%). Those who said they did, did so once a week (22.2%) or 2-3 times per week (20%). |
| Clarke (2021) | **Participant responsiveness.** Participants were asked open-ended questions about whether they enjoyed the intervention, what they liked most about the interventions and what they would change. | **Participant responsiveness.** 84% of students reported enjoying the program. When asked what they liked most about the program, participants cited (a) meditation and exercises, (b) learning about their mind set, (c) how mindful and calming the sessions were, (d) the focus on being present, (e) help overcoming everyday stress at school, and (f) the kindness and enthusiasm of the instructors. When asked what they would change about the program, while most said they enjoyed the program as it was implemented, suggestions included (a) adding relaxing music, (b) making the sessions last longer or adding more sessions, and (c) adding more stretches/exercises. |
| Frank (2021) | **Participant responsiveness.** Exploratory tests of moderation to determine whether different levels of out of class programme practice impacted outcomes. Practice was categorised into two discrete levels: practicing less than once a month (66% of intervention students) and practicing at least once a month (34% of intervention students). Moderation analyses added the interaction term between group status and programme practice and removed the main effect of programme practice **Fidelity.** Intervention fidelity (adherence to lesson components were assessed by independent coders (n=7) who were randomly assigned to code each lesson for fidelity. The intervention fidelity coding measure and manual were created by the programme developer and included point by point listing of all required actions and activities for each lesson. Videos were coded by two coders. Average inter rate agreement was 92% (disagreements resolved by third coder). | **Participant responsiveness.** More frequent program practice tended to be associated with better outcomes for a variety of measures. students in the adequate practice group showed significantly lower levels of overall difficulties in emotion regulation, lack of emotional awareness, lack of emotional clarity , impulse control difficulties, mind wandering, an significantly higher levels of social connectedness. An odds ratio of 0.75 indicating a 25% reduction in the odds of students engaging in substance abuse. To test whether practice effects were a result of pre-test difference, practice groups were compared on all pre-test self-report measures and found fewer differences than would be expected by chance (i.e. practice does not seem to be related to pre-test differences in risk. Intervention impacts on neurocognitive outcomes were not significantly moderated by program practice levels. **Fidelity.** The overall implementation fidelity across sessions was 78.60% across teachers. |
| Huppert (2010) | **Participant responsiveness:** Home practice (no. of times practiced outside of class), how much they felt they had learned during the course, how much they enjoyed the course, how helpful they found it, whether the training course was the right length and whether they thought they would continue to practice mindfulness. | **Participant responsiveness:** 33% practised at least three times a week, 34.8% practised more than once but less than three times a week and 32.7% practised once a week or less (of whom seven respondents, 8.4%, reported no practice at all). Only two students reported practicing daily. The practice variable ranged from 0 to 28 (number of days of practice over 4 weeks). The practice variable was found to be highly skewed, with 79% of the sample obtaining a score of 14 or less (skewness = 0.68, standard error of skewness = 0.25). The majority of students who experienced mindfulness training found it to be a positive experience, and 74% said they thought they would continue with the practice. Most students thought the course was about the right length, but 43% thought it should be longer. |
| Johnson (2016) | **Participant responsiveness:** Home practice: "during the 8 weeks course, how often did you practice each of the following techniques outside of the lessons? Students were supplied with a list of techniques learnt and rated each on a 5-point scale (1 “never” - 5 "3-4 timed per week". At T3 (11 weeks later) the question was reworded “Since the mindfulness course at school, how often have you used the following mindfulness techniques?” "How enjoyable/ interesting did you find the course?" "How much did you feel you learnt during the course?" (Range 0-10, higher scores = more positive). **Quality.** Rating of instructor (range 0-10, 10 = more positive). | **Participant responsiveness:**  On 0–10 Likert scales, mean scores were as follows: enjoyment and interest 6.67 (median 7, range 0–10); amount learnt 6.73 (median 7, range 0–10) and likelihood of using techniques in the future 6.14 (median 7, range 0–10). Home practice: **Quality:** Instructor rating by students for the current course was 8.49 (median 9, range 0–10). |
| Johnson (2017) | **Fidelity:** Observer rating for each lesson using adherence subscale of the Mindfulness Based Intervention - Teaching Assessment Criteria (MBI-TAC).  **Quality:** Observer rating for each lesson using **c**ompetence subscale of the MBI-TAC, participant ratings of instructor embodiment of mindfulness, coverage, pacing, organisation, and guiding mindfulness practices (range 1 - incompetent, 6 - advanced. **Participant responsiveness**. *(See Johnson 2016 for measure).* The amount of home practice was investigated as a moderator of outcome for the mindfulness group, using hierarchical multiple regression. | **Fidelity and Quality:** A score out of six was given for each of the three domains assessed, together with an overall average score for each lesson with an average in the Proficient Band (5/6) across lessons. **Participant responsiveness:** Averaged across practices and students, home practice occurred less than once a week. Homework practice did not explain any variance in depression or anxiety. Mean ratings of the course were as follows: enjoyment and interest 6.92 (median 7; range 0–10) and amount learnt 6.84 (median 7; range 0–10), comparable to those reported in earlier trials of the .b curriculum (Johnson et al., 2016, Kuyken et al., 2013). The mean reported likelihood of using mindfulness practices in the future was 6.1 (median 6; range 0–10), contrasting to the modest reported usage at six (10.6%) and twelve months follow-up (8.4%). |
| Johnson (2019) | **Participant responsiveness:**  see Johnson (2016) for measures**. Quality:** see Johnson (2016) for measure. **Fidelity.** Audio-recordings of lessons were independently rated using the MBI-TAC (see Johnson, 2016, for measure). | **Participant responsiveness:**  Of the 33 students (80.4%) at school B who returned feedback forms in the last lesson, mean ratings of the course were as follows: enjoyment and interest 6.92 (median = 7; range = 3-10), amount learnt 6.88 (median = 7; range = 4-10), and likelihood of using techniques in the future 6.59 (median = 7; range = 3-9) comparable to those reported in similar universal trials of mindfulness in secondary schools. T-tests showed no differences in these scores between Year 8 and Year 10 (enjoyment/interest, t(31) = 0.04, P = 0.97; amount learnt, t (31) = −0.07, P = 0.94; likely future use t(31) = 0.36, P = 0.72). At post-intervention (school B), only 27.1% of students undertook home practice once a week or more. At four-month follow-up, numbers of students undertaking home practice once a week or more were very low (≤ 8.0%). Mean levels of home practice did not differ across Year level during the course or at follow-up. **Fidelity and quality:**  Students (80.4%) who returned feedback forms in the last lesson, mean student ratings for the instructor were 8.9 (median = 9; range = 7-10). There were no differences in mean scores between Year 8 and Year 10 students ( t (31) = 0.73, P = 0.47). |
| Khalsa (2012) | **Dosage.** Attendance. | **Dosage.** Secondary analyses Yoga session attendance data for students in the yoga intervention group showed signiﬁcant positive correlations with the change in IPPA total score (r=0.25, p=0.041) and the change in Positive Psychological Attitudes Life Purpose and Satisfaction subscale score (r=0.26, p=0.035). There was also a signiﬁcant negative correlation between attendance and the change in Total Mood Disturbance Score on the POMS-SF (r=−0.31, p=0.012; with a signiﬁcant decrease in the subscale of Tension/Anxiety (r= −0.25, p=0.041). A signiﬁcant correlation was also observed between attendance and change in the BASC-2 subscale Attitude to School (r=−0.24, p=0.046). There were no signiﬁcant gender differences in the amount of change in study variables. |
| Kuyken (2013) | **Participant responsiveness:**  Students’ views of the acceptability of the MiSP programme were assessed at post-intervention and follow-up. Home practice: This included a general question about mindfulness practice and four questions about specific mindfulness practices taught in the MiSP curriculum. The frequency with which these practices had been used since completing the course was assessed on a six-point scale (never to almost every day). Random effects linear regression models used to test whether practice moderated the effect of the intervention on well-being, depression and stress. | **Dosage:** mean of 7.5 (out of 9) lessons (median 8, range 0–9). On ten-point Likert scales, mean enjoyment and interest was 7.0 (median 8; range 0–10). **Participant responsiveness:** 97% of participants in the intervention arm completed programme evaluations. On ten-point Likert scales, mean enjoyment and interest was 7.0 (median 8; range 0–10) and mean amount learnt was 6.6 (median 7, range 0–10). When asked to respond to the question ‘In the future, how likely are you to use the techniques you have learned?’ on a ten-point Likert scale, the mean response was 5.9 (median 6, range 0–10). 95% completed home practice measures. Around 80% of the children had used the practices to varying levels. A similar percentage of the children had focused on their breathing. Far fewer children, however, had used beditation (44%), walked a short distance or eaten a mouthful of food mindfully (52%) or noticed where in the body they were feeling stress (60%). Random effects linear regression models were run to examine whether students that showed the greatest use of mindfulness practices had better outcomes. There was evidence that those who reported more frequent home practice had better outcomes for well-being, depression and stress. |
| Lam (2020) | **Participant responsiveness:** Benefits: 13 closed-ended items on perceived benefits of the program and its major components (e.g., mindful eating/breathing; body scan). Students rated each item from 1 (not useful) to 10 (very useful). Home practice: 11 items were included to evaluate the frequency of home practice throughout the program (1 [0 times], 2[1– 2 times], 3 [3–6 times], 4 [once a week], or 5 [more than once per week]. The mean amount of practice was derived by averaging the ratings across nine specific skills taught and included in homework handouts. Utility: 9 closed-ended items scored on a 5-point Likert scale from 1 (strongly disagree) to 5 (strongly agree), such as “This group has been helpful to my school life” and “I feel better able to handle my emotions since I participated in the group.” | **Participant responsiveness:** Benefits: Marginally positive overall usefulness (M = 5.77, SD = 2.72) and satisfaction ratings (M = 6.62, SD = 2.50), and mean activity ratings ranging from 4.58 (SD = 2.25) to 5.70(SD=2.71) on a scale of 1(not useful/not satisfied) to 10 (extremely useful/extremely satisfied). The highest activity rating was for “gratitude” (M = 5.70, SD = 2.71) and the lowest was for the body scan (M = 4.58, SD = 2.25). Process evaluation showed that approximately 52.8% of participants subjectively perceived the program as useful (a rating of 6 or above on a 10-point scale), with 66% satisfied with the program (a rating of 6 or above). 15% indicated dissatisfaction with the program (a rating of below 5). Upon completion of the program, approximately 40–45% of participants agreed or strongly agreed that they had improved in managing emotions, interpersonal relationships, patience, and attention control, and 30–40% found the program helpful for their school or family life. When asked whether the program should be offered in secondary school, nearly half (41.5%) of the students agreed or agreed strongly, with another 45% not indicating a preference. 80% of students reported that they practiced the learned skills at least once or twice during the program (indicated by a rating of at least 2). 73.6% reported that they applied what they learnt to handling difficulties at least once. Spearman correlations between change scores in outcome variables and the mean amount of homework practice were non-significant. |
| Lawson (2019) | **Dosage:** Attendance (record of student absence). **Fidelity:** Both classroom teachers completed a daily fidelity checklist. The purpose of the fidelity checklist allowed the classroom teacher to date and note the events taking place throughout the study. The tool was created as a means for each teacher to make notes of students who were absent and any distractions that occurred during morning meditation in the classroom that might impact the research. | **Dosage:** Not reported. **Fidelity:** Not reported. "The researcher was actively able to ensure the session were occurring with fidelity". |
| Lombas (2019) | **Participant responsiveness:** Five items assessing the acceptability of the programme. Except for the last question whose answers were dichotomous (yes or no), the rest of the questions were rated on an 11-point scale ranging from 0 to 10, with higher scores corresponding to a higher agreement with the item | 21 students completed the questionnaire. In general, students’ scores about the questions related to the satisfaction of the programme were slightly lower to the adequate level; with scores of 4.8 or 5. Likewise, 38.1% of the students reported the wish of receiving the programme again in the future. |
| Metz (2013) | Dosage: Participation (no definition) Fidelity: Observer rated adherence checklist for 5% of sessions. Quality: Competence subscale of the MBI-TAC: 5-point scale rating of several instructor and session characteristics including (1) lesson organization and pacing, (2) management of the group process, (3) instructor ability to teach students to notice and describe their direct experience, and (4) instructor attitude, genuineness, and embodiment of mindfulness. Due to feasibility issues, few session logs were completed; however, the teacher did not report any difﬁculties with any sessions or the process. Participant responsiveness. Benefit: 10 close-ended items measuring perceived beneﬁt of the overall program and each of its components (1 - not useful, 10 - very useful. | Dosage: 100% participation rate. Quality: Participant responsiveness. The overall mean program satisfaction was high (M = 8.2, SD = 1.7, range 1-10). Higher program satisfaction was associated with improvement from pre-test to post-test in affective self-regulatory efﬁcacy, r(131)=.31, p=.000 and with increased emotional awareness, r(133)=–0.26, p=.002. Recommend programme to other (yes- 89.1%, uncertain - 7%, no - 4%). The most useful program components reported included the body scan (M =8.5, SD = 2.1), sitting mindfulness practice (M = 8.0, SD = 2.1), mindful breathing practice (M = 7.7, SD = 2.1), and mindful movement practice (M = 7.2, SD=2.5). Other program components were rated slightly lower but still of moderate value including in-class presentation (M=6.6, SD=2.2), group discussion (M=6.6, SD=2.1), practice CDs (M=6.4, SD=2.6), and workbook/handouts (M=5.1, SD=2.3). Fidelity: All observation indicated lesson adherence, teacher enthusiasm, and preparedness. |
| Mrazek (2019) | **Dosage.** Attendance | **Dosage:** On average, 80% of lessons were completed (91% for lesson 1; 82% for lesson 2; 76% for lesson 3; 69% for lesson 4). Students completed 77% of the daily exercises. In some cases, multiple classrooms were facilitated as a single group within the digital learning platform, preventing breakdown of adherence data by classroom. |
| Rice (2015) | **Fidelity:** Observer rated adherence to programme manual. **Participant responsiveness:** Observer rating of engagement for each session. Descriptors e.g. “Adolescents are engaged and compliant. They appear to complete tasks set effectively” (1 - weak evidence, 4 - strong evidence). These were converted into percentage scores by dividing mean scores by the total possible score. **Quality:** Independent rating of facilitator engagement for each session. (1 - weak evidence, 4 - strong evidence). These were converted into percentage scores by dividing mean scores by the total possible score. | **Fidelity:** manual adherence was high for all intervention (73%). **Participant responsiveness:** Observer ratings of students and facilitator engagement was good. (61%). **Quality:** Ratings of facilitator engagement were good (78%). |
| Salmoirago-Blotcher (2018) | **Dosage:** Retention, attendance. **Fidelity.** Mindfulness instructors digitally recorded each session and 10% of recordings were randomly audited. A checklist was used by health education teachers to ensure all planned topics were discussed. **Participant responsiveness:** programme satisfaction (1 - not at all, 5 - very much) | **Dosage:** Retention at intervention completion and at 6 months of follow-up was 100% and 98%, respectively. Class attendance was 96% in both conditions. Students reported listening to the recording on their own 5 (SD 2.9) times/week. **Fidelity:** Not reported. **Participant responsiveness:** >90% of students were “somewhat to very satisfied” with the health education intervention and 77% were ‘somewhat to very satisfied’ with the mindfulness program. |
| Sibinga (2016) | **Dosage:** Student Attendance. | **Dosage:** Students attended approx. 80% (74-85%) of the program sessions. |
| Worthen (2019) | **Participant responsiveness:** Home practice (participants asked to document every time they practiced outside the classroom. Effectiveness of practice (1 - not good 2 - fair 3- good 4- very good) for each programme component. | All participants received mindfulness intervention. Participants were able to choose a support strategy to complete mindfulness at home (forming 4 different conditions): (1) timetabled class + no extra support (2) timetabled class + voluntary group practice (3) timetables class + voluntary group practice + email report of total minutes practiced (4) timetabled class + daily email reminder to practice. **Participant responsiveness:** Effectiveness ratings: Body scan (M= 3.5 SD=0.64), Body scan sitting (M= 3.3 SD=0.65), Strong determination (M= 3.3 SD=0.72), Stabilising attention breath-body (M= 3.2 SD=0.64), Ice cube meditation (M= 3.2 SD= 0.5=85, ) Thought awareness (M= 3.2 SD= 0.68), Stabilising attention-breath with counting (M= 3.0 SD=0.77), Befriending loving kindness (M= 3.0 SD=81), Walking meditation (M= 2.7 SD= 0.88), Noting meditation (M= 2.6 SD=0.74). Home practice: Reports average minutes of mindfulness practice each week at post intervention for each of the 4 conditions: (1) class + no support: M= 9.1 (2) Class plus voluntary group practice: M = 11.7 (3) class + voluntary group practice + email report of total minutes practiced: M = 26.8 (4) class + daily email reminder: M= 15.6 |

**Online Resource 5.** Definitions of the theoretical model terms

| **Terms** | **Definitions** |
| --- | --- |
| *Wider context* | Wider context represents the broader structural and socioeconomic factors characterising the catchment area / district from which the schools’ students come and specifically where the school is located (Ford et al., 2020). For example, it can be operationalised along a number of different dimensions, including the country or region, an index of the area-level deprivation (e.g., Index of Multiple Deprivation, IMD), whether a school is located in a rural or urban area (urbanity), etc. |
| *School* | School, in general, refers to an establishment that educates young people. We operationally define it in terms of different aspects of the school experience (Ford et al., 2020) such as: the *characteristics of the school community*, which includes the level of school deprivation (and that can be operationalised as the proportion of students in each school who are eligible for free school meals), the level of educational needs or disabilities (that can be operationalised as the proportion of students who receive support for special educational needs or disabilities), the ethnic composition of the school (that can be operationalised as the proportion of students, identifying as ‘White’, or other ethnic groups), etc.  We also include and make a particular distinction with *operational features of the school,* which refers to characteristics of the school that can, in principle at least, be changed or intervened on. These includes, for example, the school size (that can be operationalised as the total number of students within a school), the school strategy and leadership team, resourcing, the student-to-teacher ratio, whether the school is classified as a mixed- or single-sex school, the school quality (that can be operationalised by overall school quality ratings (for example provided by the Office for Standards in Education, Ofsted), the quality of the school social-emotional learning (SEL) provision (that can be assessed using rating scales by independent assessors), the school climate quantified by means of the students’ and/or teachers’ perceptions of general aspects of school life [e.g., safety, rules, norms, values, social relationships, teaching/learning practices, school leadership, organizational structures, etc. (Thapa et al., 2013), all of which can be assessed by rating scales]. These factors can for the most part be operationally measured with relatively robust publicly available data. |
| *Teacher* | Teacher, in general, refers to someone who educates young people, in this context in schools. We operationally define the key teacher characteristics of as age, gender, ethnicity, years of teaching experience, training status (e.g., optimally trained in SBMT vs non-expert in SBMT), and mental health status (e.g., perceived stress, anxiety, depression, burnout, etc.). |
| *Students* | Students, refer to young people who are in school to study and learn. Student factors includes age, gender, ethnicity, risk for mental health problems (e.g., social-emotional-behavioural functioning, risk for depression, psychological well-being, etc.), any other health conditions. |
| *Fidelity* | Fidelity is the extent to which the intervention has been delivered in keeping with the original intended programme. This is sometimes referred to as adherence, compliance, integrity, faithful replication, etc. (Durlak & DuPre, 2008). One way that fidelity is operationalised is as the percentage of curriculum elements of the original SBMT programme that are being covered. |
| *Dose* | Dose is *how much of* the original programme has been delivered. This is sometimes referred to as quantity, intervention strength, etc. (Durlak & DuPre, 2008). This can be operationalised as the number of SBMT sessions, or the number that are attended, and may also include the length of the sessions. This can be used to calculate the exposure to a SBMT program in term of hours and minutes. |
| *Quality* | Quality refers to *how well* the programme is delivered. In other words, whether the main program was delivered clearly and correctly, as a whole and in terms of its component elements (Durlak & DuPre, 2008). One example for how this implementation variable may be operationalised is through measuring the teaching competency to deliver SBMT, as assessed by instruments like the “Mindfulness-Based Interventions – Teaching Assessment Criteria” (MBI-TAC) (Crane et al., 2012; 2013; 2020). This measure includes quality indicators that have been specifically adapted for the teaching context and can be found in https://mbitac.bangor.ac.uk/documents/MBI-TAC-for-schools.pdf |
| *Reach* | Reach refers to what proportion of the students in a school were involved in the SBMT. This is related to programme scope (universal versus indicated, for example) and the representativeness of students with respect to programme scope (Durlak & DuPre, 2008). Some operationalisations of reach include the participation rate with respect to the study population, the participation rate with respect to the wider year group (grade level), or to the whole school student population, etc. |
| *Responsiveness* | Responsiveness refers to the degree to which the programme stimulates students’ interest, holds their attention, and engages them (Durlak & DuPre, 2008). It includes aspects that may be operationalised as receptiveness, acceptability, satisfaction, enjoyment, usefulness, perceived benefits, helpfulness, intention to apply to daily life, etc., which can be measured using rating scales. |
| *Mindfulness practice* | Mindfulness practice is one of the core elements of mindfulness-based programmes, and it is posited as a major vehicle for social-emotional learning (SEL) in SBMT (Crane et al., 2017). Mindfulness practice can be guided or self-guided, as well as carried out in the context of mindfulness-based programme sessions, or outside of the prescribed programme (Kuyken et al., 2013, Stein & Witkiewitz, 2020). Both SEL broadly and SBMT specifically argue that students need to have the opportunity to learn and then practice new skills first from teacher instructions in the context of classroom sessions, and then also in non-classroom contexts, facilitating the use and generalization of new skills in everyday interactions and routines (Jones et al., 2008; Lawson et al., 2018). In the model, we refer specifically to students’ mindfulness practice outside of the prescribed programme sessions (which are normally referred to as formal practices), and it is usually assessed by self-reported measures of weekly frequency of students’ home-based mindfulness practice. However, it should not be restricted to home-based practice, as it may include specific types of practice that can be carried out in other contexts of daily life (some of them referred to as informal practices). Mindfulness practice can be broadly operationalised, for example, by referring to practices for “being mindful” in general, or it can be measured in more specific ways, for example, by asking for different types of practices, such as mindful breathing, “beditation”, walking meditation, mindful eating, noticing stress in difficult times such as exams, etc. Other potential ways of measuring mindfulness practice could incorporate the length of the specific practices, and/or involve reports by third-party evaluators using observational methods or behavioural measures. |
| *Mindfulness skills* | Mindfulness skills refer a set of inter-related psychological, behavioural, and social skills – attention, awareness, attitudes (e.g., curiosity, kindness, compassion), ability to change perspective (decentring), and skills in self-care and self-regulation (Feldman & Kuyken, 2019; Greco, Baer & Smith, 2011). Mindfulness skills can be measured by using self-report rating scales. |
| *Executive function* | Executive function refers to a broad set of cognitive processes that enable mental control to regulate one’s cognitions, emotions, and behaviours required to facilitate goal attainment (Diamond, 2013). Executive function can be measured by using established self-report rating scales. |
| *Well-being* | The World Health Organisation (WHO) defines health as “a state of complete physical, mental and social well-being and not merely the absence of disease or infirmity” (WHO, 1948). In line with this general definition of health − and following Roeser et al. (2020) to delineate which potential student outcomes could be object of interest following implementation of SBMT − the present model considers well-being as a student distal outcome that includes the following dimensions: mental health, physical health, healthy relationships, students’ behaviour and academic performance, and mindfulness and self-regulation (considered as distal outcomes). *Mental health* can be operationalised as internalising distress (e.g., stress, anxiety, depression, negative affect, rumination, somatic complains, negative coping, etc.), externalising distress (e.g., impulsivity, hyperactivity, social problems, anger, aggression, hostility, attention problems, etc), and psychological well-being (e.g., positive affect, resilience, optimism, positive self-concept, etc.)^1^. *Physical health* can be operationalised by means of physiological indicators (e.g., blood pressure, heart rate, cortisol, body mass index, etc), and behavioural indicators (e.g., sleep, health care utilization, substance use, dietary restrain, etc.). *Healthy relationships* includes prosocial skills and altruism (e.g., social skills, social-emotional competence, empathy, kindness, compassion for others, diminished affective prejudice and stereotyping, etc.), positive connections to others (e.g., cooperation, popularity, positive peer/teacher relations, positive social climate, etc.), and positive connections with nature (e.g., connection with nature and other beings, sustainable consumption, etc.). *Students’ behaviour and academic performance* includes academic motivation (e.g., academic self-concept, motivation to learn, etc.), academic behaviours (e.g., classroom engagement, rule-following, lack of disruptive behaviour, etc.), and academic performance (e.g., grades, academic skills, etc.). Finally, *mindfulness and self-regulation*, taken as distal outcomes, include mindfulness skills (e.g., mindfulness of sensation, emotion and thought, self-compassion, etc.), attention regulation (e.g., selective attention, attentional switching, working memory, self-control, inhibitory control, etc.), and emotional regulation (e.g., emotional awareness and processing, impulse control, improved cognitive reappraisal, emotional expression, positive coping, self-efficacy, etc.). |

^1^ There is agreement that, at the very least, the concept of psychological well-being includes the presence of positive emotions (e.g., contentment, happiness), the absence of negative emotions (e.g., depression, anxiety), satisfaction with life, fulfilment, and positive functioning (Andrews & Withey, 1976; Diener, 2000; Frey & Stutzer, 2002; Ryff & Keyes, 1995), so all these dimensions may be contemplated as indicators of psychological well-being.

**References for the Online Resource 4**

Andrews FM, Withey SB. (1976). Social indicators of well-being. NewYork: Plenum Press, 63–106.

Crane, R. S., et al. (2012). Competence in teaching mindfulness-based courses: Concepts, development, and assessment. *Mindfulness*, 3, 8.

Crane, R. S., et al. (2013). Development and validation of the Mindfulness-Based Interventions - Teaching Assessment Criteria (MBI:TAC). *Assessment*, 20(6), 681-688.

Crane, R. S., et al. (2017). What defines mindfulness-based programs? The warp and the weft. *Psychological medicine,* 47(6), 990–999.

Crane, R.S., et al. (2020) Assessing Mindfulness-Based Teaching Competence: Good Practice Guidance, *Global advances in Health and Medicine*, https://doi.org/10.1177/2164956120973627

Diamond A. (2013). Executive functions. *Annual review of psychology*, 64, 135–168.

Diener E. (2000). Subjective wellbeing: the science of happiness and a proposal for a national index. *American Psychologist*, 55(1):34–43.

Durlak, J. A., & DuPre, E. P. (2008). Implementation matters: a review of research on the influence of implementation on program outcomes and the factors affecting implementation. *American journal of community psychology*, 41(3-4), 327-350.

Ford, T., et al. (2021). The Role of Schools in Early Adolescents' Mental Health: Findings from the MYRIAD Study. *J Am Acad Child Adolesc Psychiatry*, 60(12), 1467–1478.

Frey BS, Stutzer A. (2002). Happiness and economics. Princeton, N.J.: Princeton University Press.

Greco, L.A., Baer, R.A., & Smith, G. (2011). Assessing mindfulness in children and adolescents: Development and validation of the child and adolescent mindfulness measure (CAMM). *Psychological Assessment*, 23. 606-614.

Jones, S. M., Brown, J. L. & Aber, J. L. (2008). Classroom settings as targets of intervention and research. In M. Shinn & H. Yoshikawa (Eds.), Toward positive youth development: Transforming schools and community programs, (pp. 58-77). New York, NY: Oxford University Press, Inc.

Kuyken, W., et al. (2013). Effectiveness of the Mindfulness in Schools Programme: non-randomised controlled feasibility study. *The British journal of psychiatry: the journal of mental science*, 203(2), 126-131.

Lawson, G. M., McKenzie, M. E., Becker, K. D., Selby, L., & Hoover, S. A. (2019). The Core Components of Evidence-Based Social Emotional Learning Programs. *Prevention science: the official journal of the Society for Prevention Research*, 20(4), 457–467.

Roeser, R.W., Galla, B.M., Baelen, R.N. (2020). Mindfulness in Schools: Evidence on the Impacts of School-Based Mindfulness Programs on Student Outcomes in P-12 Educational Settings. Retrieved January 5, 2021, from https://www.prevention.psu.edu/uploads/files/PSU-Mindfulness-Brief-0223.pdf

Ryff CD, Keyes CLM (1995). The structure of psychological well-being revisited. *Journal of Personality and Social Psychology*, *69*(4):719–727.

Stein, E., Witkiewitz, K. (2020). Dismantling Mindfulness-Based Programs: a Systematic Review to Identify Active Components of Treatment. *Mindfulness* *11*, 2470–2485.

Thapa A, et al. (2013). A Review of School Climate Research. *Rev Educ Res*, *83*(3), 357-385.

World Health Organization. Regional Office for Europe. (‎1984)‎. Health promotion : a discussion document on the concept and principles : summary report of the Working Group on Concept and Principles of Health Promotion, Copenhagen, 9-13 July 1984. Copenhagen : WHO Regional Office for Europe. https://apps.who.int/iris/handle/10665/107835
